# Cytoskeletal Dynamics in Cancer: From Pathogenesis to Treatment

**DOI:** 10.1002/mco2.71016

**Published:** 2026-09-25

**Authors:** Jie Chen, Wenxi Yang, Yonghan Song, Yuanhao Zhang, Jia Ma, Tingyao Wang, Jijun Zheng, Hebin Zhang, Yiyao Liu, Jinhao Zeng

**Affiliations:** ^1^ Department of Gastroenterology Hospital of Chengdu University of Traditional Chinese Medicine Chengdu China; ^2^ Traditional Chinese Medicine (TCM) Prevention and Treatment of Metabolic and Chronic Diseases Key Laboratory of Sichuan Province Hospital of Chengdu University of Traditional Chinese Medicine Chengdu Sichuan China; ^3^ School of Life Science and Technology University of Electronic Science and Technology of China Chengdu China

**Keywords:** actin filaments, cancer, cytoskeletal dynamics, intermediate filaments, microtubules

## Abstract

Cancer remains a major global health burden despite recent advances in early diagnosis and novel therapeutics. The cytoskeleton is a dynamic filamentous network that controls cell morphology, motility, and intracellular trafficking; in normal cells, its activity is tightly regulated, whereas in cancer, it undergoes aberrant remodeling that fuels uncontrolled proliferation, metastatic dissemination, resistance to apoptosis, and epithelial–mesenchymal transition. These pathological changes are associated with Rho guanosine triphosphatases (Rho GTPases) cascades, Hippo/Yes‐associated protein (YAP) signaling, and other mechanosensitive pathways. However, a comprehensive understanding of how cytoskeletal dynamics integrate with these signaling networks and the tumor microenvironment to drive malignant progression and modulate treatment responses is still lacking. This review systematically addresses this gap by dissecting the diverse contributions of the cytoskeleton to tumor proliferation, invasion, programmed cell death, and angiogenesis, and by clarifying how mechanical signals synergize with cytoskeletal rearrangements to enhance aggressive behaviors. It further evaluates the modulatory effects of cytoskeletal components on the efficacy of conventional chemotherapy, immunotherapy, and molecular targeted therapy, and summarizes the clinical evidence for microtubule‑stabilizing and destabilizing drugs. Taken together, this study offers fresh perspectives on the intricate interplay between the cytoskeleton and cancer, and provides an important reference for the current field of cancer research.

## Graphical abstract

This review systematically addresses the fundamental roles of the cytoskeleton (actin filaments, microtubules, and intermediate filaments) in cancer progression. We focus on how cytoskeletal dynamics regulate tumor proliferation, metastasis, and programmed cell death, while also modulating the immune microenvironment. Understanding these mechanisms may guide targeted therapy development and ultimately improve clinical outcomes for patients.

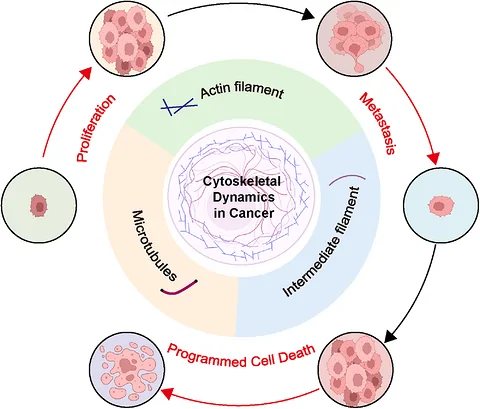

## 1 Introduction

Cancer is a disease caused by the loss of normal regulation and subsequent excessive proliferation of the somatic cells. Its core hallmarks are genetic and epigenetic alterations in somatic cells, which drive sustained proliferative signaling, cell death, angiogenesis, and metastasis [1, 2]. Cancer has become one of the leading causes of human death globally. Based on the latest projections, approximately 20 million individuals worldwide were diagnosed with cancer for the first time in 2022. Lung cancer ranks as the leading cause of cancer‑related mortality, while other frequently diagnosed malignancies comprise colorectal, prostate, gastric, hepatic, and cervical cancers [3, 4, 5, 6]. Carcinogenesis and cancer progression are driven by the interplay of multiple factors including environmental exposure, lifestyle, and chronic infection [7, 8].

Cytoskeletal architecture plays a pivotal role in the morphological and functional transformations observed during cancer progression. Comprising microtubules, actin filaments, and intermediate filaments, the cytoskeleton orchestrates cellular integrity, polarity, and dynamic behavior [9]. Actin filaments, assembled from globular actin (G‐actin) into filamentous actin (F‐actin), regulate cell motility and contractility through interactions with Myosin II, forming a force‐generating actomyosin network [10]. Rho family GTPases serve as key regulators of actin cytoskeletal assembly and reorganization, modulating not only cytoskeletal dynamics but also vesicular trafficking, cell polarity, enzyme activity, and transcriptional responses [11]. Microtubules, composed of α‐ and β‐tubulin heterodimers, facilitate intracellular transport, structural support, and directional migration [12]. Their dynamic instability, characterized by rapid transitions between polymerization and depolymerization, is essential for cell cycle progression. Intermediate filaments, noted for their mechanical stability, serve as scaffolding elements that integrate and anchor microtubules and actin filaments, thereby maintaining cytoskeletal cohesion and structural resilience [13]. Moreover, the cytoskeleton plays a pivotal role in determining the mechanical properties and mechanotransduction of cells. Its network architecture defines the physical properties of cells, such as elasticity and stiffness, and can convert mechanical signals including extracellular matrix (ECM) stiffness, stress, and compressive stress into intracellular biochemical signals. This regulates cell proliferation, apoptosis, tissue homeostasis maintenance, immune response, and injury repair [14].

Conventional cancer therapies, including chemotherapy, radiotherapy, surgery, and immunotherapy, have significant limitations [15, 16]. In recent years, drug synthesis research targeting the cytoskeleton, particularly microtubules, has increased dramatically [17]. Microtubule‑targeting agents (MTA) constitute an important class of therapeutic agents in cancer treatment [18]. Their mechanism of action primarily relies on perturbing the dynamic equilibrium of microtubules during cell division, thereby arresting the cell cycle and ultimately inducing cell death. Two principal categories exist for these agents based on how they affect microtubules: stabilizing drugs (taxanes) and destabilizing drugs (vinca alkaloids). Their widespread clinical adoption notwithstanding, the survival outlook for many patients remains grim [19]. Currently, research on novel MTA and other cytoskeleton‑targeted therapeutic approaches is ongoing, with the aim of reducing side effects. Therefore, in‑depth exploration of the molecular mechanisms of the cytoskeleton in cancer development is crucial for the development of new drugs and the investigation of novel therapies.

Aberrant remodeling of cytoskeletal proteins contributes to tumor initiation, progression, and therapeutic resistance. The process of metastatic dissemination, fueled by dysregulated cell motility, relies heavily on cytoskeletal reorganization [20]. Elucidating the roles of cytoskeletal dynamics in cancer may reveal critical molecular events and enable the identification of actionable targets. Recent advances in targeting cytoskeletal components and associated signaling pathways have yielded promising pharmacological agents, with mechanistic insights informing the development of novel interventions against cancer pathogenesis. This study begins with an overview of cytoskeletal composition and dynamics, covering microfilaments, microtubules, intermediate filaments, and their associated proteins. It then discusses the roles of the cytoskeleton in tumor proliferation, metastasis, and programmed cell death, with a focus on its involvement in differential protein expression and signaling pathway regulation. In addition, the study examines how mechanical stiffness and forces influence tumor progression. Finally, the therapeutic potential of targeting the cytoskeleton is discussed, encompassing conventional therapy, immunotherapy, and targeted therapy. This study systematically synthesizes the past decade's advances regarding the cytoskeleton in cancer, providing an important reference for the current field of cancer research.

## 2 The Components and Dynamics of Cytoskeleton

**FIGURE 1:**
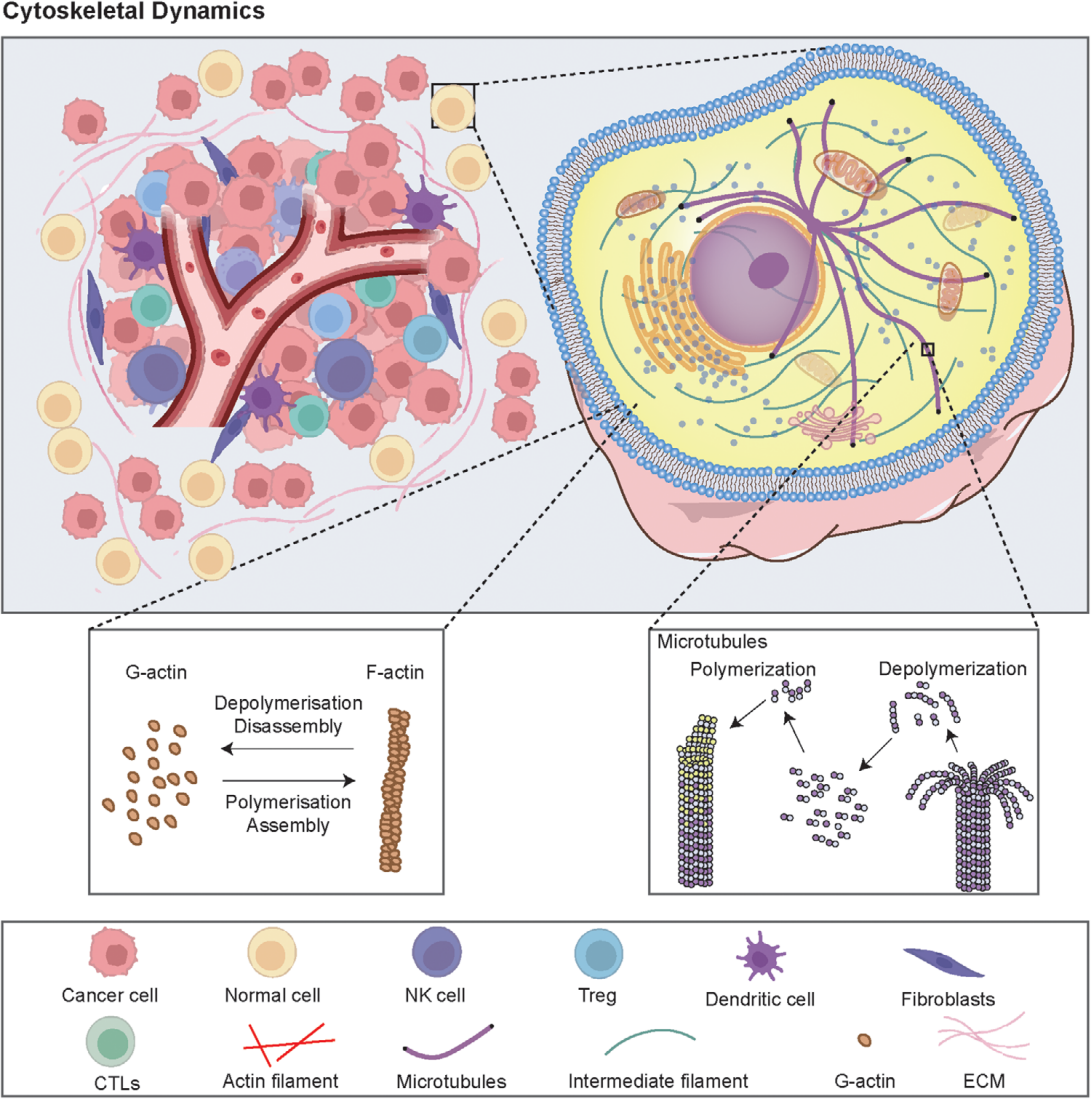
Components and dynamics of the cytoskeleton. Comprising microtubules, actin filaments, and intermediate filaments, the cytoskeleton orchestrates cellular integrity, polarity, and dynamic behavior.

The cytoskeleton, a structurally and functionally versatile framework in eukaryotic cells, orchestrates critical cellular processes such as cell division, motility, and response to external stimuli. These processes are mediated by three primary types of cytoskeletal filaments: microfilaments (actin filaments), microtubules, and intermediate filaments. Together, these filamentous structures form a dynamic network (Figure 1) [21].

### 2.1 Actin Filaments

Actin filaments are composed of actin monomers, which exist in two forms: G‐actin and F‐actin. As the most dynamic of the cytoskeletal proteins, actin enables rapid structural remodeling within minutes, facilitating changes in cell shape. G‐actin polymerizes into asymmetric, helical F‐actin structures, typically extending to 6–7 µm in vitro [22]. These filaments regulate numerous cellular functions, including motility and structural adaptation. The elongation and stabilization of actin filaments are controlled by actin‐binding proteins, which govern polymerization, depolymerization, and cross‐linking dynamics [23].

Rho GTPases, members of the Ras superfamily, are pivotal in cell migration signaling pathways. Ras‐related C3 botulinum toxin substrate 1 (Rac1), cell division control protein 42 (Cdc42), and RhoA are the prototypical GTPases in this family [24], with well‐documented roles in regulating actin cytoskeleton organization and dynamics. In fibroblasts, Rac1 induces lamellipodia and ruffles, Cdc42 promotes filopodia formation, and RhoA drives stress fiber assembly [25]. These structures are crucial for actin‐driven protrusion at the leading edge, mediated by Rac1/Cdc42‐induced lamellipodia and filopodia, while RhoA‐dependent stress fibers regulate actomyosin contraction at the trailing edge.

Rac1 activates the Wiskott‐Aldrich syndrome protein‐family verprolin‐homologous protein (WAVE) complex to stimulate actin‐related protein (Arp) 2/3‐mediated actin polymerization at lamellipodia. Both Rac1 and Cdc42 interact with p21‐activated kinase (PAK), activating it and downstream substrates like LIM kinase (LIMK), which enhances actin polymerization through cofilin phosphorylation. Additionally, Rac1 and Cdc42 bind to phosphatidylinositol 3‐kinase (PI3K), and PI3K‐phosphorylated lipids stimulate Rac1 guanine nucleotide exchange factors (GEFs), creating a positive feedback loop that amplifies cell motility. Cdc42 also recruits the WASP–Arp2/3 complex to nucleate actin bundles in filopodia. RhoA, through Rho kinases (ROCK1/2), regulates actomyosin contractility and focal adhesion (FA) maturation [26].

Cofilin mediates actin filament severing and barbed‐end generation, accelerating both depolymerization and polymerization cycles [27]. This activity provides G‐actin for cytoskeletal remodeling, enabling the formation of lamellipodia, pseudopodia, and filopodia—structures critical for tumor cell chemotaxis and invasion. Cofilin also enhances Arp2/3‐mediated dendritic nucleation by producing fragmented actin filaments [28].

### 2.2 Microtubules

Microtubules, originating from microtubule‐organizing centers, primarily the centrosomes, consist of α‐, β‐, and γ‐tubulin subtypes [29]. These dynamic polymers form intracellular transport networks for vesicles, organelles, and macromolecules. Their stability and behavior are regulated by microtubule‐associated proteins (MAPs), which can exert stabilizing or destabilizing effects, influencing polymerization rates or promoting microtubule severing.

MAPs are a class of proteins that specifically bind to microtubules. Their main functions include promoting microtubule assembly, enhancing microtubule structural stability, mediating intracellular transport, and regulating microtubule dynamics. A large and diverse range of MAPs have been identified to date, such as nucleolar and spindle‐associated protein 1 (NUSAP1), discs large‐associated protein 5 (DLGAP5), protein regulator of cytokinesis‐1 (PRC1), microtubule‐associated protein tau (MAPT), hepatoma upregulated protein (HURP), and never in mitosis gene A‐related kinase 2 (NEK2). MAPs exhibit cell‐ and tissue‐specific expression and play important roles in cancer. Microtubule‐stabilizing proteins including tau and MAP2 inhibit disassembly and facilitate microtubule elongation. In contrast, microtubule‐destabilizing proteins promote depolymerization. For example, stathmin (STMN) promotes depolymerization by binding to tubulin heterodimers. Furthermore, posttranslational modifications of microtubules, including acetylation, can influence their stability by modulating MAP activity, thereby controlling microtubule dynamics.

### 2.3 Intermediate Filaments

Intermediate filaments, a diverse protein family encoded by over 70 genes, assemble into 10‐nm‐diameter filaments. These filaments are classified into five types based on sequence homology: cytoplasmic intermediate filaments (Types I–IV) include keratins (KRTs) (Types I/II), vimentin (Type III), desmin (Type III), and neurofilaments (Type IV), while nuclear lamins (Type V) are located in the nucleus [30]. Cytoplasmic intermediate filaments form a dense reticular network, primarily positioned in the perinuclear space, extending to the cortex [31]. These filaments bind to local adhesion sites to maintain cell adhesion [32].

Intermediate filaments are critically involved in counteracting external mechanical stress. Vimentin establishes a compact perinuclear framework that safeguards the nucleus from such forces. Furthermore, vimentin strengthens cellular attachment to viscoelastic environments and accelerates cell spreading. The interaction between vimentin and actin filaments is critical for cell adhesion, migration, and invasiveness. KRTs are mainly present in all epithelial cells and are classified into Type I (acidic) and Type II (basic) families. Different types of KRTs are expressed in various parts of the human body, tissues, and cells. KRTs help maintain cell shape and resist external stress, providing structural support.

### 2.4 Cytoskeleton‐Related Research Methods

A comprehensive understanding of cytoskeletal dynamics in cancer depends heavily on the methodological tools available to visualize and quantify actin, microtubule, and intermediate filament organization, and the choice of imaging modality substantially shapes the biological conclusions that can be drawn. Conventional confocal laser scanning microscopy and total internal reflection fluorescence microscopy remain widely used for characterizing cytoskeletal architecture in fixed and living cells, owing to their relative accessibility and compatibility with standard immunofluorescence and live‐cell labeling protocols. However, their diffraction‐limited resolution of roughly 200–250 nm is insufficient to resolve the nanoscale filament bundling, protofilament arrangement, and adhesion‐complex organization that are frequently altered during malignant transformation, meaning that subtle but functionally important remodeling events can be missed or misinterpreted using these approaches alone [33].

To overcome this limitation, super‐resolution imaging strategies have been increasingly applied to cytoskeletal research in recent years. Stochastic optical reconstruction microscopy has been directly used to resolve nanoscale differences in actin bundle width and tubulin organization between mesenchymal stem cells and cancer cell lines, including glioblastoma and breast cancer cells, revealing cell‐type‐specific cytoskeletal remodeling patterns that are not detectable by conventional fluorescence microscopy and that may correlate with differences in invasiveness or lineage identity [34]. Advances in 4Pi‐detection single‐molecule localization microscopy have further pushed axial resolution to the range of a few nanometers, enabling near‐isotropic three‐dimensional visualization of cytoskeletal filament networks that was previously inaccessible with standard two‐dimensional super‐resolution approaches, an advance particularly relevant for resolving how cytoskeletal architecture is organized across the full cell volume during processes such as spindle assembly and mitotic remodeling [35]. In parallel, quantitative structured illumination microscopy (SIM) methods incorporating physical model‐based background‐filtering algorithms have improved the accuracy of live‐cell actin dynamics imaging by suppressing reconstruction artifacts common to conventional SIM processing, allowing more reliable quantification of filament turnover and remodeling kinetics in real time rather than relying on static snapshots [36].

Beyond hardware‐ and algorithm‐based reconstruction methods, deep‐learning‐based image restoration has emerged as a complementary strategy for enhancing spatial resolution and signal‐to‐noise ratio while minimizing phototoxicity and photobleaching, both of which are major constraints for prolonged live‐cell imaging of dynamic cytoskeletal processes; systematic evaluations of such neural‐network‐based approaches have demonstrated their capacity to substantially improve image quality across diverse fluorescence microscopy platforms without requiring additional photon exposure [37].

Collectively, the integration of high‐resolution and super‐resolution imaging modalities, three‐dimensional localization methods, and advanced quantitative and deep‐learning‐based reconstruction algorithms has become indispensable for dissecting the fine structural and dynamic changes of the cytoskeleton that underlie tumor initiation, invasion, and therapeutic response, thereby providing an important methodological foundation for studying cytoskeleton‐related mechanisms.

## 3 Cytoskeletal Dynamics in Cancer Pathogenesis

The cytoskeleton, a dynamic and interconnected filamentous network, is critical for regulating cell shape, division, and motility. Dysregulation of the cytoskeleton promotes cancer progression by inducing proliferation, metastasis, programmed cell death, and angiogenesis [38].

### 3.1 Cytoskeletal Dynamics in Cancer Proliferation

Cytoskeletal dynamics play a crucial role in cancer proliferation. This role is primarily manifested in multiple aspects, including metabolic reprogramming, differential protein expression, signaling pathway regulation, and cell cycle control, significantly affecting the growth and survival of cancer cells. Cytoskeletal dynamics related to cancer proliferation are summarized in Table 1.

**TABLE 1:** Summary of identified targets and proposed mechanisms linking cytoskeletal dynamics to cancer cell proliferation.

Cytoskeletal structure	Main contributor	Target/mechanism	Cancer type	References
Actin‐binding protein	Cofiln‐1	Lactylation	Nasopharyngeal carcinoma	[41]
Microtubule	LC3	NAT10/HK2 axis	Gastric cancer	[42]
Cytoskeleton	KRT6A	MYC‐regulated pentose phosphate pathway	Lung cancer	[43]
Actin‐binding protein	TAGLN	p53 acetylation	Glioblastoma	[44]
Actin‐binding protein	AVIL	−	Rhabdomyosarcoma	[45]
Actin‐capping proteins	CAPZA1	−	Cancer	[46]
Actin‐binding protein	Ezrin	Actin cytoskeleton	Pancreatic ductal adenocarcinoma	[47]
Microtubule‐associated protein	NUSAP1	−	Pan‐cancer	[48]
Microtubule	MTUS1	DNA methylation	Non‐small cell lung carcinoma	[49]
Microtubule	α‐Tubulin	Acetylation	Cervical cancer	[50]
Cytoskeleton	KRT19	Histone deacetylation	Liver cancer	[51]
Cytoskeleton	ACTNs	Hippo signaling and Rho GTPase	Hepatocellular carcinoma	[54]
Actin‐binding protein	DBN1	NF2–LATS kinases complex	Liver cancer	[55]
Microtubule	α‐Tubulin	YAP	Non‐small‐cell lung cancer	[56]
Microtubule	MAST3	YAP phosphorylation	Breast cancer	[57]
Focal adhesion complex	Zyxin	YAP	Colon cancer	[58]
Microtubule‐associated protein	DLGAP5	Wnt/β‐catenin signaling pathway	Endometrial cancer	[59]
Microtubule‐associated protein	PRC1	Wnt/β‐catenin signaling pathway	Lung adenocarcinoma	[60]
Actin filament associated protein	AFAP1‐AS1	Wnt/β‐catenin signaling pathway	Lung adenocarcinoma	[62]
Actin‐binding protein	ACTN4	ACTN4–RIPK1–NF‐κB signaling axis	Melanoma	[63]
Actin cytoskeleton	Actin	β‐Catenin	Colorectal cancer	[65]
Microtubule	GEF‐H1	GEF‐H1/RhoA signaling pathways	Breast cancer	[66]
Actin filament associated protein	AFAP1‐AS1	−	Retinoblastoma	[67]
Actin filament associated protein	AFAP1‐AS1	Ras/MEK/c‐Jun and cadherin/vimentin signaling pathways	Hepatocarcinoma	[68]
Actin‐binding protein	Girdin	JAK/STAT signaling pathway	Colorectal carcinoma	[69]
Cytoskeleton‐associated protein	CKAP5	YAP	Esophageal squamous	[75]
Actin‐binding protein	CLP	IL‐24/MAPK/ERK/TGFβ signaling pathway	Breast cancer	[76]
Actin‐binding protein	ANLN	miR‐218‐5p/LASP1 signaling pathway	Pancreatic cancer	[77]
Actin‐binding protein	ENAH	PI3K/AKT signaling	Laryngocarcinoma	[78]
Cytoskeleton	Actin	G_0_/G_1_ cell cycle arrest	Breast cancer	[80]
Cytoskeleton	CKAP2L	G2/M phase	Pan‐cancer	[81]
Actin‐binding protein	Anillin	Cytokinesis	Hepatocellular carcinoma	[82]
Actin‐binding protein	ANLN	Cell cycle arrest at the G2/M phase	Breast cancer	[83]
Actin‐binding protein	FGD4	Cell cycle arrest at the G2/M phase	Prostate cancer	[84]
Actin filament‐associated protein	AFAP1‐AS1	Cell cycle arrest at the G2/M phase	Pancreatic cancer	[85]
Intermediate filament	KRT80	Cell cycle arrest at the G1 phase	Colon cancer	[86]
Microtubule	α‐Tubulin	Cell cycle arrest at the G2/M phase	Esophageal squamous cell carcinoma	[87]
Microtubule‐associated protein	MAP9	Mitotic defects	Colorectal cancer	[88]
Microtubule	Stathmin	Cell cycle arrest at the G2/M phase	Glioblastoma	[89]
Microtubule‐associated protein	PRC1	Cell cycle arrest at the G2/M phase	Hepatocellular carcinoma	[90]

*Abbreviations*: ACTNs, α‐actinins; AFAP1‐AS1, actin filament‐associated protein 1 antisense RNA 1; ANLN, anillin; AVIL, advillin; CAP1, cyclase‐associated protein 1; CKAP2, cytoskeleton‐associated protein 2; CLP, coactosin‐like protein; DBN1, drebrin; DLGAP5, discs large‐associated protein 5; ENAH, enabled homolog; FGD4, frabin; GEFs, guanine nucleotide exchange factors; KRTs, keratins; LC3, microtubule‐associated protein 1 light chain 3 alpha; MAPs, microtubule‐associated proteins; MAST3, microtubule‐associated serine/threonine kinase‐3; MTUS1, microtubule‐associated tumor suppressor 1; NUSAP1, nucleolar and spindle‐associated protein 1; PRC1, protein regulator of cytokinesis‐1; TAGLN, transgelin.

#### 3.1.1 Metabolic Reprogramming

**FIGURE 2:**
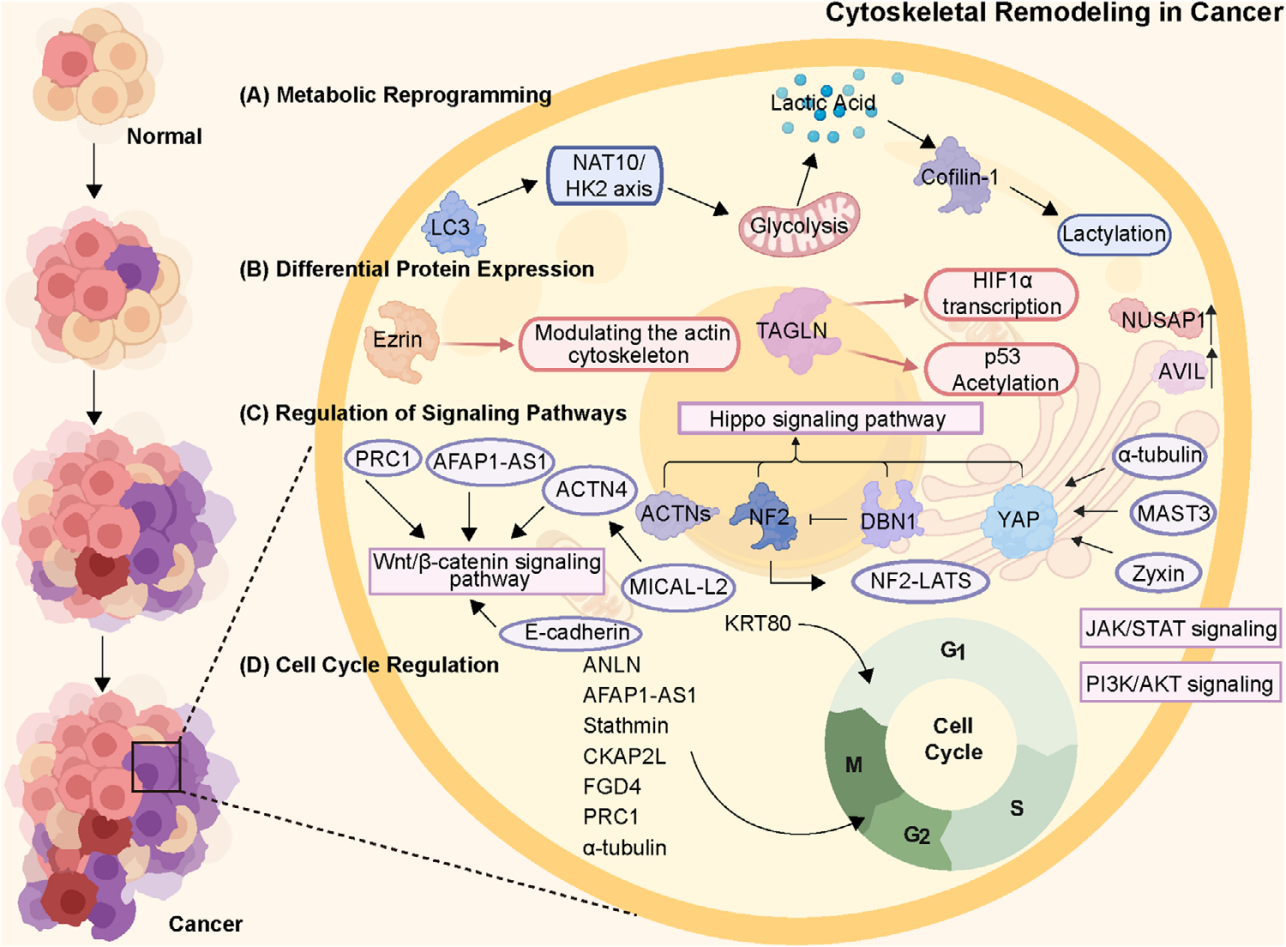
Cytoskeletal remodeling in cancer proliferation. (A) Metabolic reprogramming. LC3 is involved in the degradation of NAT10, which drives glycolytic metabolism and gastric carcinogenesis. Enhanced glycolysis leads to the production and accumulation of lactate. Elevated lactate levels promote the lactylation of the actin‐binding protein Cofilin‐1 at Lysine 22, thereby promoting the proliferation of nasopharyngeal carcinoma cells. (B) Differential protein expression. Ezrin regulates the actin cytoskeleton to influence cell morphology, thereby playing a role in the growth of pancreatic ductal adenocarcinoma. TAGLN regulates HIF1α transcription and stabilizes HDAC2 to deacetylate p53, thereby promoting cancer stem cell survival. NUSAP1 and AVIL is highly expressed in various tumor tissues and can significantly promote cell proliferation. (C) Regulation of signaling pathways. ACTNs promote hepatocellular carcinoma via Hippo inhibition and Rho GTPase activation. DBN1 disrupts the NF2–LATS1/2 complex, driving liver tumorigenesis. YAP interacts with α‑tubulin during mitosis. MAST3, downregulated in breast cancer, promotes YAP phosphorylation and degradation. Zyxin facilitates colon cancer growth via CDK8‑mediated YAP activation. PRC1, AFAP1‐AS1, ACTN4, MICAL‑L2, and E‑cadherin drive tumor progression by activating Wnt/β‑catenin signaling. JAK/STAT signaling pathways and PI3K/AKT signaling pathways are also involved in regulating cell proliferation. (D) Cell cycle regulation. KRT80 knockdown leads to G1 arrest. ANLN, AFAP1‐AS1, CKAP2L, FGD4, PRC1, and the microtubule regulators (acetylated α‑tubulin and Stathmin) affect G2/M progression.

The glycolytic pathway is particularly important during metabolic reprogramming of tumor cells (Figure 2A). Cancer cells exhibit a strong dependence on aerobic glycolysis, which not only sustains the urgent energy demands for rapid proliferation but also provides essential biosynthetic precursors [39]. Beyond its canonical role in energy production, a growing body of evidence indicates that glycolytic flux and its byproducts actively crosstalk with the cytoskeletal architecture to coordinate malignant progression. On one hand, the tumor microenvironment directly couples glucose metabolism to cytoskeletal mechanics. Hyperglycemic conditions modulate the cytoskeletal architecture of breast cancer cells via the ROCK and focal adhesion kinase (FAK) signaling pathways, thereby altering their mechanical elasticity [40]. Elevated lactate levels promote the lactylation of Cofilin‐1 at Lysine 22, thereby promoting the proliferation of nasopharyngeal carcinoma cells [41]. Conversely, cytoskeletal regulators and metabolism are mutually influential. For instance, the microtubule‐associated protein 1 light chain 3 alpha (LC3) drives glycolytic metabolism and gastric carcinogenesis through the degradation of N‐acetyltransferase 10 (NAT10) [42], KRT6A upregulates the expression of glucose‐6‐phosphate dehydrogenase, consequently boosting the metabolic throughput of the pentose phosphate pathway [43]. In short, this mutual regulation means the cytoskeleton does more than just respond to metabolism. It actively controls key metabolic steps.

#### 3.1.2 Differential Protein Expression

Upregulation or downregulation of cytoskeleton‑associated proteins is closely linked to cancer development (Figure 2B). Extensive evidence indicates that malignant transformation is consistently accompanied by substantial dysregulation of multiple such proteins, and this aberrant expression endows cancer cells with enhanced proliferative capacity. Based on their structural classes, these cytoskeleton‑associated proteins can be categorized into three major groups, namely, actin‑binding proteins, microtubule‑associated proteins, and intermediate filaments proteins, each contributing to tumor progression through distinct mechanisms.

Among actin‑binding proteins, transgelin (TAGLN) acts under hypoxia by modulating hypoxia‐inducible factor 1alpha (HIF1α) transcription and histone deacetylase‐2 (HDAC2)‑mediated p53 deacetylation to sustain cancer stem cell survival [44], while advillin (AVIL) is markedly overexpressed in rhabdomyosarcoma [45]. The capping proteins CAPZA1/CAPZB restrict branched F‑actin around multivesicular bodies, thereby promoting their peripheral secretion and tumor‑associated intercellular communication [46], and ezrin remodels cortical actin architecture to influence pancreatic ductal adenocarcinoma cell morphology and growth [47]. For microtubule‑associated proteins, NUSAP1 drives proliferation across multiple cancer types [48], whereas the tumor suppressor microtubule‑associated tumor suppressor 1 (MTUS1) is epigenetically silenced in non‑small cell lung carcinoma, correlating with enhanced proliferation [49]. Additionally, primary cilia are microtubule‐based organelles that process various metabolic and extracellular signals. Inhibition of α‐tubulin acetylation shortens cilia length, thereby accelerating the progression of cervical cancer [50]. With respect to intermediate filaments, KRT19 participates in histone deacetylation and liver tumorigenesis, and has also been implicated in cancer stem cell reprogramming, drug sensitivity modulation, and lung cancer initiation and progression [51, 52, 53].

#### 3.1.3 Regulation of Signaling Pathways

Signaling pathways is intimately associated with the development and persistence of various tumor types (Figure 2C). Modulating these pathways can profoundly influence tumor cell behavior. Among the numerous cascades, the Hippo/yes‑associated protein (YAP) pathway is a classic axis that controls cancer cell proliferation. Several cytoskeletal proteins have been shown to modulate this pathway through distinct mechanisms. For instance, α‑actinin (ACTN) proteins, which are major cytoskeletal components, are significantly upregulated in hepatocellular carcinoma (HCC) and act as tumor promoters by inhibiting Hippo signaling while elevating Rho GTPase activity [54]. Similarly, the actin‑binding protein drebrin (DBN1) directly interacts with neurofibromin 2 (NF2), a component of the Hippo pathway, thereby disrupting the NF2–LATS1/2 complex formation and subsequently promoting liver tumorigenesis [55]. YAP itself participates in mitosis mainly through its interaction with α‑tubulin [56]. Conversely, microtubule‑associated serine/threonine kinase 3 (MAST3), which is expressed at low levels in breast cancer cells and correlates with poor prognosis in advanced tumors, interacts with YAP and promotes its phosphorylation, leading to protease‑mediated degradation of YAP [57]. Additionally, zyxin, a FA component involved in actin filament polymerization, promotes colon cancer cell growth through cyclin‐dependent kinase 8 (CDK8)‑mediated YAP activation [58]. Collectively, these findings highlight the diverse ways in which cytoskeletal proteins interface with the Hippo/YAP pathway to drive tumor progression.

Beyond the Hippo/YAP axis, the Wnt/β‑catenin signaling pathway plays a key regulatory role in cell proliferation. Multiple cytoskeleton‑associated proteins have been implicated in its activation. For example, DLGAP5 enhances the malignant behavior of endometrial cancer cells by activating Wnt/β‑catenin signaling [59], and PRC1 similarly promotes lung adenocarcinoma progression through the same cascade [60, 61]. Actin filament associated protein 1 antisense RNA 1 (AFAP1‐AS1) is involved in regulating the Wnt/β‐catenin signaling pathway in lung adenocarcinoma [62]. At the protein level, actinin‐4 (ACTN4) promotes β‑catenin expression, thereby increasing the transcription of proliferation‑related genes, and has been shown to stimulate proliferation of both melanocytes and melanoma cells [63]. In kidney clear cell carcinoma (KIRC), molecules interacting with CasL‐Like 2 (MICAL‐L2) is overexpressed and accelerates cancer progression by interacting with ACTN4 in a Rab13‑dependent manner [64]. Furthermore, E‑cadherin connects to the actin through its interaction with β‑catenin in tumor progression [65].

In parallel, Rho signaling pathways are involved in a broad range of biological processes, including cytoskeletal remodeling, cell proliferation, and migration. GEF‐H1 is a microtubule‐associated activator of RhoA. Activation of the GEF‐H1/RhoA signaling pathway promotes the proliferation of breast cancer cells and influences tumor formation [66]. Additionally, AFAP1‑AS1, which correlates with tumor size, accelerates cellular carcinogenesis [67]. AFAP1‑AS1 drives HCC cell proliferation via the Ras/mitogen‐activated protein kinase (MEK)/c‑Jun signaling cascade [68].

Moreover, several other signaling cascades also contribute to the regulation of cell proliferation in a cytoskeleton‑dependent manner. Overexpression of the actin‑binding protein girdin activates the Janus kinase (JAK)/signal transducer of activation (STAT) pathway, thereby promoting colorectal cancer cell proliferation [69]. Cytoskeleton‑associated protein 2 (CKAP2) promotes gastric cancer cell proliferation [70]. CKAP4, in turn, is associated with cell proliferation in gliomas, lung cancer, and esophageal cancer [71, 72, 73, 74], while CKAP5 promotes esophageal squamous cell carcinoma (ESCC) development by regulating microtubule dynamics and YAP activation [75]. In contrast, among actin‑binding proteins, coactosin‑like protein (CLP) inhibits breast carcinoma proliferation via transforming growth factor beta (TGF‐β)/SMAD pathway blockade [76], while anillin (ANLN) facilitates pancreatic tumor advancement by modulating the enhancer of zeste homolog 2 (EZH2)/miR‑218‑5p/LIM and SH3 protein 1 (LASP1) cascade [77], and enabled homolog (ENAH) stimulates PI3K/AKT signaling to accelerate laryngeal cancer progression [78].

#### 3.1.4 Cell Cycle Regulation

Cell cycle regulation is fundamental to tumor cell proliferation (Figure 2D). Throughout the cell cycle, cells actively reorganize the actin cytoskeleton and alter nuclear morphology [79]. Aberrant regulation of this process leads to uncontrolled proliferation, making a thorough understanding of the underlying mechanisms essential for elucidating tumor growth.

The actin cytoskeleton plays a pivotal role in cell cycle progression. Disruption of actin organization can halt cell motility and induce G0/G1 arrest [80]. Several actin‑binding or actin‑associated proteins have been shown to modulate specific cell‑cycle transitions. For instance, CKAP2L, a cell cycle‑related protein highly expressed across multiple cancers and linked to the tumor immune microenvironment, drives progression from G2 to M phase, thereby enhancing proliferative capacity [81]. ANLN, which regulates cytokinesis and enhances tumor growth in HCC [82], and it also plays an important role during cell division in breast cancer cells, leading to G2/M accumulation [83]. Frabin (FGD4), an actin‑binding protein involved in cytoskeletal reorganization, is upregulated in prostate cancer; its knockdown reduces proliferation and causes G2/M arrest [84]. Similarly, inhibition of AFAP1‑AS1 leads to G2/M arrest and blocks pancreatic cancer cell proliferation [85]. In contrast, reduced expression of KRT80 induces G1 arrest in colon cancer cells, accompanied by a decrease in the G2/M population [86].

Microtubules also play a critical role in cell cycle regulation. Downregulation of acetylated α‑tubulin severely disrupts microtubule maturation, inhibits assembly, and ultimately leads to G2/M arrest in ESCC cells [87]. MAP9, a microtubule‑stabilizing agent that mediates mitotic spindle assembly, when silenced, causes severe mitotic defects and promotes colorectal cancer development [88]. STMN is involved in microtubule polymerization, and its downregulation leads to G2/M arrest in glioblastoma cells [89]. Furthermore, inhibition of PRC1 expression also induces G2/M arrest in lung adenocarcinoma cells [60], while overexpression of PRC1 drives HCC tumor progression and relieves G2/M checkpoint blockade through the p21/p27/pRB cascade [90].

Taken together, the cytoskeleton plays a critical role in tumor cell proliferation, thereby driving cancer progression. In‑depth studies on the cytoskeleton have not only enhanced our understanding of cancer biology but also provided potential therapeutic targets for the development of novel treatment strategies.

### 3.2 Cytoskeletal Dynamics in Cancer Metastasis

Invasion and metastasis constitute a principal hallmark of the cancerous phenotype. The role of cytoskeletal dynamics in cancer metastasis has attracted considerable attention. Cytoskeletal dynamics influence cancer metastasis through multiple mechanisms, including epithelial–mesenchymal transition (EMT), differential protein expression, the immune microenvironment, and signaling pathways. Cytoskeletal dynamics related to cancer metastasis are summarized in Table 2.

**TABLE 2:** Summary of cytoskeletal dynamics‐associated molecules and mechanisms in cancer metastasis.

Cytoskeletal structure	Main contributor	Target/mechanism	Cancer type	References
Intermediate filament	Vimentin	CAF	Lung adenocarcinoma	[92]
Intermediate filament	Vimentin	DNA nonhomologous end joining repair	Cancer	[93]
Intermediate filament	KRT8	EMT	Gastric cancer	[95]
Intermediate filament	KRT17	EMT	Gastric cancer	[96]
Actin‐binding protein	Ezrin	E‐cadherin	Cholangiocarcinoma	[97]
Actin‐binding protein	TAGLN	Invadopodia formation and EMT	Bladder cancer	[98]
Actin filament cross‐linking protein	FLNA	EMT	Colon cancer	[99]
Actin cytoskeleton	ARHGAP29	RhoA/ROCK signaling pathway	Melanoma	[100]
Actin cytoskeleton	ARHGAP10	PI3K/Akt/GSK3β signaling pathway	Non‐small cell lung cancer	[101]
Actin‐binding protein	Cofilin‐1	RhoA–LIMK2–Cofilin‐1 signaling pathway	Colorectal cancer	[102]
Microtubule‐associated protein	MAP4	GSK3β/β‐catenin signaling pathway	Hepatocellular cancer	[103]
Cytoskeletal tropomyosin	TPM2	Fascin‐1	Cancer	[104]
Microtubule	STMN2	TGF‐β signaling pathway	Hepatocellular carcer	[105]
Microtubule	STMN2	WNT/β‐catenin signaling pathway	Pancreatic cancer	[106]
Microtubule	STMN1	E‐cadherin	Hypopharyngeal squamous cell carcinoma	[107]
Actin‐binding protein	Cortactin	The Arp 2/3 complex	Head and neck squamous cell carcinoma	[110]
Actin‐related protein	Arp2/3	TGF‐β signaling pathway	Breast cancer	[111]
Actin‐related protein	Arp2/3	TGF‐β signaling pathway	Pancreatic ductal adenocarcinoma	[112]
Actin‐binding protein	EPLIN	Rab21 endosomes	Breast cancer	[114]
Actin‐binding protein	Palladin	Actin polymerization	Cancer	[115]
Cytoskeleton	ACTN	Alternative splicing	Glioblastoma	[116]
Actin‐regulating protein	CAP1	Phosphorylation	Pancreatic cancer	[117]
Microtubule	GEF‐H1	RhoA signaling pathway	Breast cancer	[118]
Cytoskeleton	ARHGEF7	Actin cytoskeleton	Colorectal adenocarcinoma	[122]
Intermediate filaments	Vimentin	Actin stress fibers and podosomes	Lung adenocarcinoma	[124]
Actin‐binding protein	LSP1	−	Cervical cancer	[125]
Microtubule‐associated protein	NEK2	AKT signaling pathway	Hepatocellular carcinoma	[127]
Microtubule	Stathmin	RhoA/ROCK signaling pathway	Neuroblastoma	[131]
Actin dynamics‐related protein	SCIN	RhoA/FAK signaling pathway	Glioma	[132]
α‐tubulin	ATAT1	RhoA signaling pathway	Breast cancer	[133]
Actin‐binding protein	Cofiln‐1	P38 MAPK signaling pathway	Prostate cancer	[135]
Microtubule	STMN1	P38 MAPK signaling pathway	Non‐small cell lung cancer	[136]
Actin‐binding protein	TWF2	Hippo signaling pathway	Renal cell carcinoma	[137]
Intermediate filament	IFFO1	IQGAP3–Cdc42	Lung cancer	[138]
Microtubule‐associated protein	MAP4	The ERK–c‐Jun–vascular endothelial growth factor A signaling pathway	Esophageal squamous cell carcinoma	[140]

Abbreviations: ACTNs, α‐actinins; ARHGAP10, Rho GTPase activating protein 10; ARHGEF7, Rho guanine nucleotide exchange factor 7; Arp, actin‐related protein; CAP1, cyclase‐associated protein 1; EPLIN, epithelial protein lost in neoplasm; FLNA, filamin A; GEFs, guanine nucleotide exchange factors; KRTs, keratins; LSP1, leukocyte‐specific protein 1; MAPs, microtubule‐associated proteins; NEK2, never in mitosis gene A‐related kinase 2; SCIN, scinderin; STMN, stathmin; TAGLN, transgelin.

#### 3.2.1 Epithelial–Mesenchymal Transition

**FIGURE 3:**
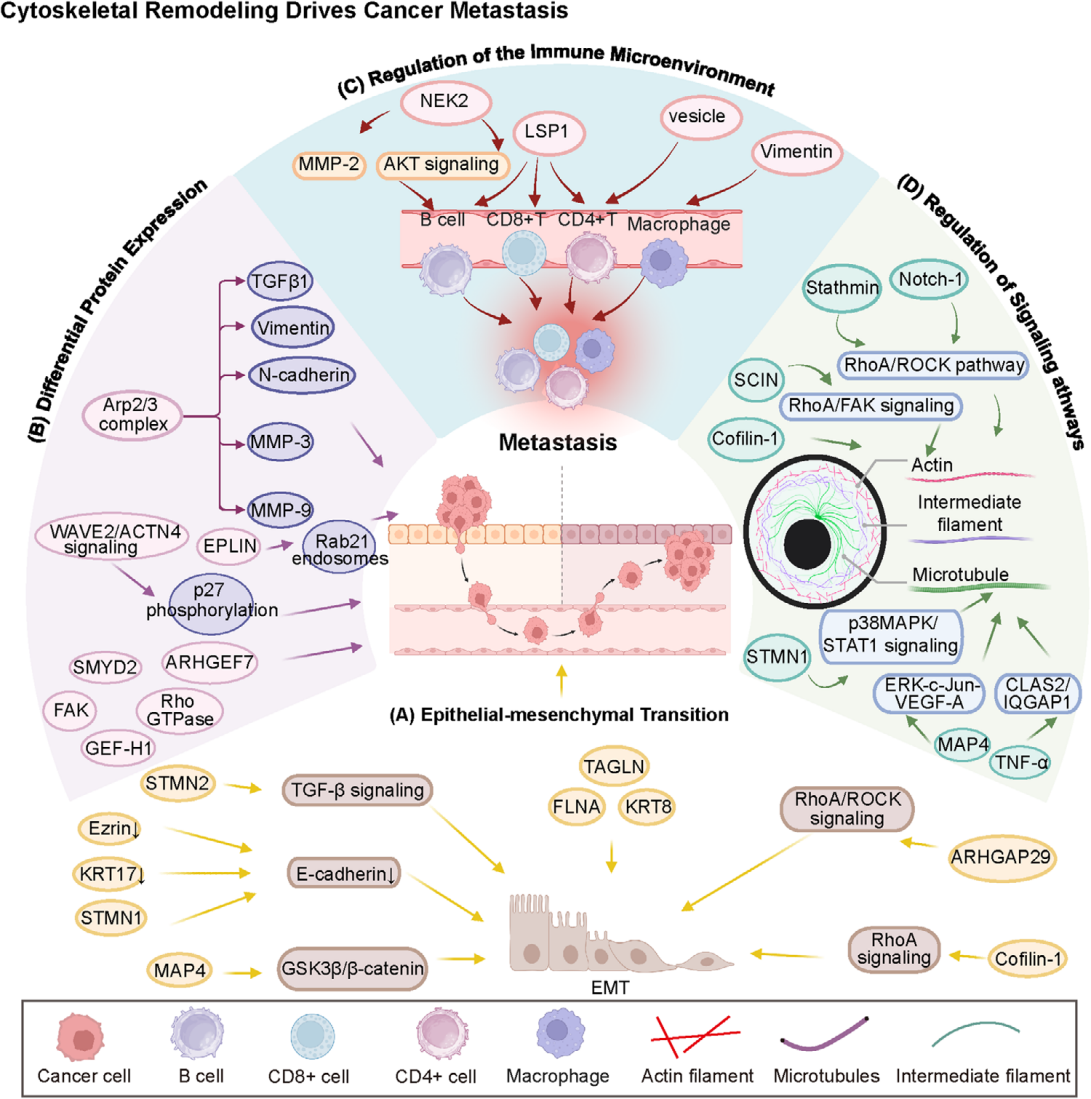
Cytoskeletal remodeling drives cancer metastasis. (A) Epithelial–mesenchymal transition. Ezrin links membrane proteins to actin; its downregulation promotes E‐cadherin internalization and migration in cholangiocarcinoma. ARHGAP29, MAP4, STMN1, and STMN2 promote EMT in various cancers. Cofilin‑1 regulates actin organization through RhoA–LIMK2–Cofilin‑1 signaling to modulate EMT in colon cancer. TAGLN drives EMT and invadopodia formation in bladder cancer. FLNA correlates with Snail‑induced EMT. KRT8 promotes proliferation/migration via integrin β1–FAK‑dependent EMT; KRT17 loss induces EMT and metastasis. (B) Differential protein expression. The Arp2/3 complex activates TGF‑β signaling, upregulates N‑cadherin, vimentin, MMP‑9, MMP‐3, and promotes metastasis. WAVE2/ACTN4 signaling enhances pancreatic cancer motility via p27 phosphorylation. EPLIN drives breast cancer migration by regulating integrin recycling from Rab21 endosomes. Microtubule dynamics regulate contractility and focal adhesion maturation through Rho GTPase and GEF‐H1. FAK reorganizes actin to promote migration. ARHGEF7 regulates actin cytoskeleton and is linked to colorectal metastasis. SMYD2‑induced cytoskeletal remodeling drives breast cancer metastasis. (C) Regulation of the immune microenvironment. Vimentin coordinates actin stress fibers and podosomes to promote macrophage‑mediated degradation of the ECM and lung cancer invasion. LSP1, an actin‑binding protein expressed in TIM‑3‑low CD4^+^/CD8^+^ T cells, correlates with activated B and T cell abundance. Microtubule dynamics regulate vesicle transport and immunosuppressive cytokine secretion, affecting T cell function. NEK2 drives HCC invasion/metastasis via AKT/MMP‑2 and supports marginal zone B cell development. (D) Regulation of signaling pathways. Stathmin promotes neuroblastoma metastasis via RhoA/ROCK signaling and transendothelial migration independently of tubulin; Notch‑1 also activates RhoA/ROCK to drive F‑actin remodeling and contractility. SCIN activates RhoA/FAK to promote glioma migration/invasion. Cofilin‑1 drives prostate cancer migration/invasion through p38 MAPK‑mediated actin remodeling. STMN1 promotes migration via microtubule stabilization, and also via p38 MAPK/STAT1 independently of microtubules. IFFO1 negatively regulates lung cancer metastasis by inhibiting IQGAP3–Cdc42 interaction. TNF‑α induces CLASP2/IQGAP1‑dependent cytoskeletal remodeling to drive bladder cancer metastasis. MAP4 promotes ESCC invasion/migration via ERK–c‑Jun–VEGF‑A signaling. Wnt/β‑catenin activation correlates with adrenocortical carcinoma invasiveness, coordinating cytoskeletal reorganization and signaling to promote metastasis.

EMT represents a typical event in the initial phase of tumor metastasis (Figure 3A). During this process, the cytoskeleton undergoes profound reorganization: microtubules adopt a more radial arrangement with accelerated plus‑end growth, actin stress fibers align in parallel with the long axis of the cell, while FAs shrink in size [91].

Several cytoskeletal proteins directly contribute to EMT and subsequent migration. Among intermediate filaments, vimentin serves as both a hallmark of EMT and a key driver of metastasis. It facilitates lung adenocarcinoma metastasis by maintaining interactions between heterogeneous cancer cells and fibroblasts [92], and it also regulates lipid metabolism via adipose triglyceride lipase, thereby playing a role in inhibiting metastasis under DNA damage conditions [93]. Epithelial cell migration requires KRT [94]. KRT8 overexpression enhances proliferation and migration in gastric cancer, and integrin β1–FAK‑induced EMT occurs only in cells with high KRT8 expression [95]. Conversely, downregulation of KRT17 induces E‑cadherin loss, triggers EMT, and promotes metastatic behavior in gastric cancer cells [96]. In addition, actin‑binding proteins modulate EMT through distinct mechanisms. Ezrin maintains epithelial integrity by interacting with E‑cadherin; its downregulation during cholangiocarcinogenesis leads to E‑cadherin internalization and enhanced cell migration [97]. TAGLN, which is highly expressed in metastatic bladder cancer, promotes cell migration by inducing EMT [98]. Filamin A (FLNA) upregulation correlates with EMT and altered cell adhesion [99].

Beyond these direct structural regulators, multiple signaling pathways orchestrate actin cytoskeleton dynamics, induce EMT, and promote cancer invasion. The Rho/ROCK axis is a prominent mediator. For instance, Rho GTPase activating protein (ARHGAP)29 promotes EMT via the RhoA/ROCK pathway [100], whereas ARHGAP10, which is expressed at low levels in non‐small cell lung cancer (NSCLC), inhibits EMT by inactivating the PI3K/Akt/GSK3β pathway [101]. Cofilin‑1, another key regulator, modulates actin organization through the RhoA–LIMK2–Cofilin‑1 cascade, thereby affecting EMT in colon cancer cells [102]. The PI3K/Akt pathway is also targeted by other proteins. MAP4 promotes EMT in HCC by regulating the GSK3β/β‑catenin signaling axis [103]. Interestingly, tropomyosin isoforms encoded by TPM2 regulate fascin‑1 activity, thereby inhibiting cancer metastasis [104]. The Wnt/β‑catenin pathway is another target. STMN2, which is upregulated in HCC, coordinates microtubule depolymerization via the TGF‑β pathway to promote EMT [105], and in pancreatic ductal adenocarcinomas (PDAC), it activates Wnt/β‑catenin signaling, inducing EMT and altering Cyclin D1 expression [106]. Similarly, STMN1 promotes EMT by downregulating E‑cadherin [107]. Finally, the Notch1 pathway exerts a counteracting effect. Its activation induces cytoskeletal remodeling, enhances cell–cell junctions and cell–matrix adhesion, and prevents GSK‑3β phosphorylation and β‑catenin nuclear translocation, thereby upregulating E‑cadherin and strengthening tight junctions, ultimately inhibiting cell migration [108].

#### 3.2.2 Differential Protein Expression

Dysregulation of the actin cytoskeleton enhances the motility and invasiveness of tumor cells (Figure 3B). A number of actin‑binding proteins and MAPs have been implicated in this process, each contributing through distinct molecular mechanisms.

Among these proteins, cortactin plays a central role in cell migration and is overexpressed in many solid tumors [109]. Cortactin promotes the formation of branched actin networks by activating the Arp2/3 complex. This activation drives cell migration and increases degradation of the ECM through the regulation of invadopodia formation and activity [110]. Moreover, the Arp2/3 complex significantly upregulates the expression of mesenchymal markers such as matrix metalloproteinase (MMP)‑9, MMP‑3, N‑cadherin, and vimentin, while also activating the TGF‑β signaling pathway, thereby promoting EMT [111]. In PDAC, the Arp2/3 complex is involved in metastasis, a process that is associated with TGFβ1 [112]. Another group of actin regulators, the WAVE family proteins, also activate the Arp2/3 complex to stimulate actin polymerization. In particular, WAVE2 signaling, in coordination with ACTN4, selectively stimulates p27 phosphorylation, thereby enhancing the invasiveness of pancreatic cancer cells [113].

In addition to the Arp2/3‑related pathways, several other actin‑binding proteins directly modulate cell migration. Epithelial protein lost in neoplasm (EPLIN) drives breast cancer cell migration by regulating integrin recycling from Rab21 endosomes [114]. Palladin accelerates actin polymerization and is associated with cancer metastasis [115]. Filamins (FLNs) and ACTNs are both linked to cell migration, and dysregulation of their alternative splicing further enhances migratory capacity [116]. Cyclase‐associated protein 1 (CAP1) may regulate the invasiveness of pancreatic cancer cells through its effects on actin filament turnover [117].

Beyond the actin cytoskeleton itself, microtubules and FAs also play critical roles in cell migration. During migration, microtubules undergo dynamic turnover, which influences FA maturation via Rho GTPase regulation. The interaction between microtubules and the actomyosin network is governed by GEF‑H1, which is set free either when microtubules depolymerize or when it separates from FAs [118]. Cell migration further depends on the formation of FAs [119, 120], and FAK drives actin cytoskeleton reorganization to promote cancer cell migration [121]. Additionally, Rho guanine nucleotide exchange factor 7 (ARHGEF7) is associated with colorectal adenocarcinoma metastasis, and its function involves the regulation of actin cytoskeletal dynamics [122]. SET and MYND domain‐containing protein 2 (SMYD2) methyltransferase‑induced cytoskeletal remodeling has been shown to drive breast cancer metastasis [123].

#### 3.2.3 Regulation of the Immune Microenvironment

Cytoskeletal dynamics significantly affects tumor immune evasion and metastatic potential by altering immune cell function and infiltration (Figure 3C). This process plays a critical role in mediating the interaction between tumor cells and immune cells.

Several cytoskeletal components have been implicated in modulating specific immune cell types. Vimentin coordinates actin stress fibers and podosomes, thereby promoting macrophage‑mediated degradation of the ECM. This degradation subsequently enhances the invasive ability of lung cancer cells [124]. Leukocyte‐specific protein 1 (LSP1) is mainly localized in CD4^+^ and CD8^+^ T cells, and is positively correlated with the abundance of activated B cells, CD8^+^ T cells, and CD4^+^ T cells [125]. Additionally, the density and dynamics of the microtubule network regulate vesicle transport, which is associated with the secretion of immunosuppressive cytokines, thereby affecting the function of effector T cells [126].

Beyond T cells and macrophages, other cytoskeletal regulators influence B cell development and tumor metastasis. For instance, altered expression levels of NEK2 may contribute to the metastasis of HCC, possibly through the activation of AKT signaling and the promotion of MMP‑2 expression [127]. NEK2 serves a critical function in marginal zone B‑cell development, and additionally, it may be involved in the formation of conventional B cells. Furthermore, cancer cells can directly remodel the actin cytoskeleton to impair natural killer (NK) cell activation. This remodeling promotes the recruitment of inhibitory signals to the immunological synapse, thereby suppressing NK cell function.

#### 3.2.4 Regulation of Signaling Pathways

Signaling pathways plays a critical role in tumor cell metastasis (Figure 3D). The cytoskeleton can influence tumor cell behavior through multiple signaling cascades, most notably the Rho pathway and the mitogen‐activated protein kinases (MAPK) pathway, among others.

During metastasis, the ECM is broken down by cancer cells through the formation of actin‑enriched protrusions called invadopodia. The formation of these structures is regulated by Rho GTPases [128]. Among these, the Rho/ROCK axis stands out as a key regulator of cell motility. It modulates the cytoskeleton by stabilizing actin filaments and promoting the formation of stress fibers [129]. For example, activation of the RhoA/ROCK1 axis promotes actin cytoskeleton remodeling, thereby enhancing the metastatic ability of gastric cancer cells [130]. STMN mediates neuroblastoma metastasis through RhoA/ROCK signaling, and this effect occurs in a tubulin‑independent manner [131]. Additionally, Notch‑1 signaling activates the RhoA/ROCK pathway, leading to F‑actin reorganization and myosin‑mediated contractile force generation [108].

Beyond the core Rho/ROCK axis, other proteins modulate this pathway to influence metastasis. RhoA itself controls actin filament organization and stress fiber formation, and its inhibition can paradoxically enhance cancer cell invasion. Scinderin (SCIN) regulates the actin by activating the RhoA/FAK signaling pathway, thereby promoting the migration of glioma cells [132]. Disruption of alpha‐Tubulin acetyltransferase 1 (ATAT1) downregulates microtubule acetylation and consequently inhibits RhoA expression [133]. Moreover, the inflammatory cytokine IL‑1β promotes cancer cell metastasis by activating RhoA signaling, which induces actin remodeling and pseudopodia formation [134].

In addition to the Rho pathway, other signaling cascades also contribute to tumor cell migration through cytoskeletal regulation. Cofilin‑1 promotes F‑actin cytoskeleton remodeling and enhances the migration of prostate cancer cells by activating the p38 MAPK signaling pathway [135]. STMN1 promotes cell migration by regulating microtubule stability. Additionally, it can promote cell migration through activation of the p38 MAPK/STAT1 signaling pathway in a manner independent of microtubule stability [136]. As an actin‐binding protein, Twinfilin 2 interacts with YAP and prevents its degradation, thereby inhibiting the Hippo signaling pathway and promoting renal cell carcinoma (RCC) metastasis [137]. In contrast, the intermediate filament protein IFFO1 inhibits the IQ Motif Containing GTPase Activating Protein 3 (IQGAP3)–Cdc42 interaction in a dose‑dependent manner, thereby negatively regulating lung cancer cell metastasis [138]. TNF‐α promotes cytoplasmic linker associated protein 2 (CLAS2)/IQGAP1‐dependent cytoskeletal remodeling, thereby driving bladder cancer metastasis [139]. MAP4 promotes the migration of ESCC cells by activating the ERK–c‐Jun–VEGF‐A signaling pathway [140]. Activation of the Wnt/β‐catenin signaling pathway is associated with the invasiveness of adrenocortical carcinoma. Activated tumor cells reorganize the cytoskeletal network and coordinate signaling pathways to promote tumor cell metastasis [141].

Taken together, the cytoskeleton promotes cancer metastasis via diverse molecular mechanisms. Systematic elucidation of these mechanisms will not only deepen our knowledge of the metastatic process but also provide important insights for the design of novel therapeutic approaches.

### 3.3 Cytoskeletal Dynamics in Programmed Cancer Cell Death

Cytoskeletal dynamics play a crucial role in programmed cell death in cancer, influencing tumor cell survival and death through various mechanisms. The regulation of signaling pathways enables cytoskeletal dynamics to exert effects on apoptosis, autophagy, and disulfidptosis, among others. Cytoskeletal dynamics related to cancer cell death are summarized in Table 3.

**TABLE 3:** Mechanistic pathways linking cytoskeletal dynamics to programmed cell death and angiogenesis in cancer.

Consequence	Cytoskeletal structure	Main contributor	Target/mechanism	Cancer type	References
Apoptosis	Intermediate filaments	CK18	FAS and FADD	Cervical cancer	[142]
Apoptosis	Intermediate filament	KRT8	Annexin A2	Anaplastic thyroid carcinoma	[143]
Apoptosis	Intermediate filament	KRT8/18	FAS	Granulosa cell tumors	[144]
Apoptosis	Microtubule‐binding protein	NUSAP1	−	Renal cell carcinoma	[145]
Apoptosis	Microtubule‐associated protein	MAP9	ERCC3	Hepatocellular carcinoma	[146]
Apoptosis	Microtubule‐associated protein	MASTL	DNA damage	Thyroid cancer	[147]
Apoptosis	Microtubule	STMN1	−	Lung squamous cell carcinoma	[148]
Apoptosis	Microtubule	STMN1	p38 MAPK kinase and p53/p21 signaling pathway	Gallbladder carcinoma	[149]
Autophagy	Microtubule	LC3	Methylation and degradation	Ovarian cancer	[150]
Autophagy	Microtubule‐associated protein	Tau	Autophagosome–lysosome fusion	Hepatocellular carcinoma	[151]
Autophagy	Actin cytoskeleton	CLDN6	JNK/c‐Jun signaling pathway	Breast cancer	[152]
Autophagy	Actin‐binding protein	TWF1	cAMP signaling pathway	Lung adenocarcinoma	[153]
Autophagy	Intermediate filament	Vimentin	Rictor/AKT/β‐catenin signaling pathway	Cancer	[154]
Autophagy	Microtubule‐associated protein	MAP7	−	Cervical cancer	[155]
Disulfidptosis	Actin cytoskeleton	DRGs	DNA methylation	Pan‐cancer	[156]
Disulfidptosis	Actin cytoskeleton	CD2AP	NADPH and actin network	Hepatocellular carcinoma	[157]
Anoikis	Intermediate filament	Vimentin	Integrin β1	Cancer	[158]
Angiogenesis	Actin cytoskeleton	ADD3	VEGF–VEGFR‐2	Glioblastoma	[160]
Angiogenesis	Intermediate filament	Vimentin	Inflammation	Cancer	[161]
Angiogenesis	Cytoskeleton associated protei	CKAP4	EGFL6/CKAP4/ERK axis	Colorectal cancer	[162]

Abbreviations: ADD3, adducin 3; CD2AP, CD2‐associated protein; CKAP, cytoskeleton‐associated protein; ENAH, enabled homolog; KRTs, keratins; LC3, microtubule‐associated protein 1 light chain 3 alpha; MAPs, microtubule‐associated proteins; MAST, microtubule‐associated serine/threonine kinase; NUSAP1, nucleolar and spindle‐associated protein 1; STMN, stathmin.

#### 3.3.1 Apoptosis

**FIGURE 4:**
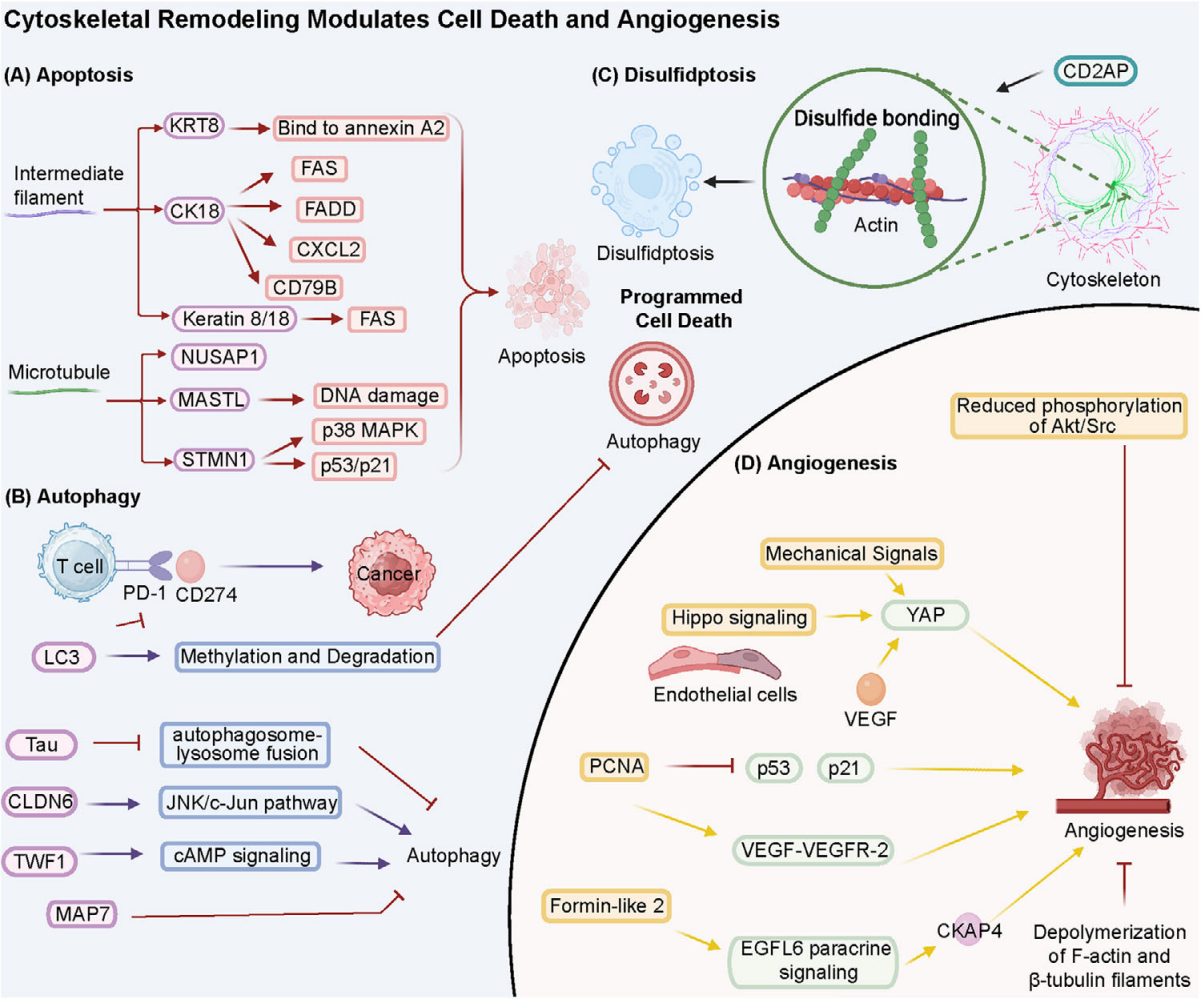
Cytoskeletal remodeling modulates cell death and angiogenesis. (A) Cytoskeletal dynamics in apoptosis. CK18 is cleaved by caspases during apoptosis and transcriptionally regulates apoptotic (FAS, FADD) and immune‑related (CXCL2, CD79B) genes. KRT8 binds to annexin A2, confers resistance to apoptosis under oxidative stress, and together with KRT18 reduces FAS expression to exert antiapoptotic effects in granulosa cell tumors. NUSAP1 depletion inhibits migration, proliferation, and invasion, and induces apoptosis in renal cell carcinoma. MAP9 ectopic expression triggers apoptosis and cell cycle arrest. MASTL depletion enhances DNA damage leading to apoptotic cell death. STMN1 inhibition reduces proliferation/invasion, increases apoptosis and paclitaxel sensitivity; its silencing modulates p38 MAPK/p53/p21 signaling and induces G2/M delay and apoptosis in gallbladder cancer cells. (B) Cytoskeletal dynamics in autophagy. LC3 mediates autophagosome formation; it can degrade CD274 via autophagy–lysosomal pathway to counter immune evasion. LC3B methylation promotes its ubiquitination/degradation, reducing autophagy and exerting protumor effects in ovarian cancer. Tau blocks autophagosome–lysosome fusion to promote tumor growth. CLDN6 regulates autophagy via JNK/c‐Jun pathway. Twinfilin 1 (TWF1) interacts with p62 and participates in autophagy regulation through the cAMP signaling pathway. MAP7 promotes cervical cancer migration, invasion, and EMT through autophagy regulation. (C) Cytoskeletal dynamics in disulfidptosis. CD2AP promotes tumor progression by regulating disulfidptosis. (D) Cytoskeletal dynamics in angiogenesis. YAP promotes endothelial proliferation, migration, and sprouting in response to VEGF and ECM stiffness. ADD3 downregulation in GBM enhances angiogenesis via PCNA‑mediated p53/p21 suppression and VEGF–VEGFR‑2 signaling. Formin‑like 2 regulates colorectal cancer angiogenesis through exosomal EGFL6/CKAP4 signaling. Reduced Akt/Src phosphorylation and F‑actin/β‑tubulin depolymerization collectively impair neovascularization.

Apoptosis can be triggered by internal and external signals that involve various enzymes (such as caspases) and diverse signaling pathways (Figure 4A). During epithelial cell apoptosis, cytokeratin 18 (CK18) undergoes caspase‐mediated cleavage. In addition, CK18 regulates the transcription of the apoptosis‐related genes FAS and FADD, as well as the immune‐related genes CXCL2 and CD79B [142]. Under peroxide‐induced cellular stress conditions, KRT8 overexpression confers resistance to apoptosis [143]. KRT 8/18 confers antiapoptotic ability in granulosa cell tumors by reducing FAS expression [144]. The expression of NUSAP1 is relatively elevated in RCC. Depletion of NUSAP1 inhibits RCC cell proliferation and induces apoptosis [145]. Ectopic expression of MAP9 in cancer cells induces apoptosis and cell cycle arrest [146]. Depletion of MASTL is associated with enhanced DNA damage, ultimately leading to apoptosis [147]. Lung squamous cell carcinoma (LSCC) cells with STMN1 inhibition exhibit reduced proliferation and invasion abilities, while their apoptotic capacity and sensitivity to paclitaxel are increased [148]. Silencing of STMN1 may regulate p53/p21 signaling pathways. Knockdown of STMN1 induces apoptosis in gallbladder cancers (GBC) cells and delays G2/M phase transition [149].

#### 3.3.2 Autophagy

Autophagy is a cellular process involving the degradation and recycling of intracellular components, thereby maintaining cellular homeostasis (Figure 4B). LC3 participates in the formation of autophagosomes and is closely related to cellular autophagy. After binding to the PD‐1 receptor, CD274 can help tumor cells evade T‐cell immune surveillance. Posttranslational modifications of LC3 are essential for regulating its function. Methylation of LC3B promotes its ubiquitination and degradation, leading to reduced autophagosome formation, thereby exerting a protumor effect in ovarian cancer [150]. MAPT also promotes xenograft tumor growth by blocking autophagosome–lysosome fusion [151]. Claudin‐6 (CLDN6) regulates autophagy through WASP‐interacting protein (WIP)‐dependent actin cytoskeleton [152]. Twinfilin 1 interacts with p62 and participates in autophagy [153]. The absence of vimentin averts autophagy‑dependent Rictor turnover by curtailing AMP‐activated protein kinase (AMPK)‑directed autophagic signals [154]. MAP7 overexpression in cervical cancer promotes EMT via autophagy control [155].

#### 3.3.3 Disulfidptosis and Others

Disulfidptosis is defined as extensive disulfide bonding to actin cytoskeletal proteins, leading to actin contraction and cytoskeletal disruption, thereby causing cell death (Figure 4C) [156]. CD2‐associated protein (CD2AP) is an actin cytoskeleton‐associated protein that promotes tumor progression by regulating disulfidptosis [157]. Vimentin boosts integrin β1 expression and triggers integrin‑dependent clustering, preventing anoikis [158]. Taken together, the cytoskeleton is involved in the regulation of programmed cancer cell death via various molecular mechanisms.

### 3.4 Cytoskeletal Dynamics in Angiogenesis

Angiogenesis plays a critical role in tumor progression (Figure 4D). YAP is a key regulator of this process. In endothelial cells, activated YAP promotes proliferation, migration, and sprouting. YAP activity is modulated by both biochemical signals, such as vascular endothelial growth factor (VEGF), and mechanical signals [159].

Several other cytoskeleton‑associated proteins also influence angiogenesis through distinct mechanisms. Adducin 3 (ADD3) is an important assembly factor in the actin cytoskeleton, and its expression is downregulated in glioblastoma multiforme (GBM). In ADD3‑depleted GBM cells, proliferating cell nuclear antigen (PCNA) promotes proliferation and angiogenesis while suppressing p53 and p21 expression. This process induces proangiogenic signaling through VEGF–VEGFR‑2‑mediated endothelial cell activation [160]. Extracellular vimentin exerts proangiogenic effects by functionally mimicking VEGF, but it also acts as an inhibitor of leukocyte–endothelial interactions [161]. Formin‑like 2 promotes epidermal growth factor‐like protein (EGFL6) paracrine signaling via exosomes to regulate angiogenesis in colorectal cancer, and CKAP4 serves as a downstream target of EGFL6 and participates in this process [162]. In addition, depolymerization of actin and β‑tubulin filaments and reduced phosphorylation of Akt/Src collectively lead to decreased neovascularization [163].

In summary, the cytoskeleton is broadly involved in cancer progression at various stages through multiple molecular mechanisms, encompassing key processes such as tumor proliferation, metastasis, cell death, and angiogenesis.

## 4 Mechanics and Signaling: How Stiffness and Force Shape Tumor Progression

Solid tumors are characterized by an abnormal mechanical microenvironment, in which the finely tunable actin cytoskeleton governs cell mechanics and thus constitutes a central intervention point. Mechanical forces, such as stiffness and stress, have been shown to influence cell growth. Cells primarily sense and respond to various mechanical forces by regulating the cytoskeleton [164]. Hence, mechanical forces should be considered an essential component of the tumor microenvironment.

### 4.1 Stiffness

Stiffness is a key component of the mechanical microenvironment and is commonly used to describe a material's ability to resist deformation. Different tissues exhibit significantly different stiffness characteristics. For instance, brain tissue is relatively soft, whereas bone tissue has high stiffness. Pathological processes such as inflammation, fibrosis, and tumors can also alter the composition and mechanical properties of the ECM, thereby forming an aberrant mechanical microenvironment [165]. Matrix stiffness is not only a structural feature of tissues but also serves as an important biomechanical signal that regulates cell behavior.

ECM stiffness‑based mechanotransduction is widely recognized as a signaling mechanism operating across healthy and pathological settings. It connects the tumor ECM to the actin cytoskeleton, thereby enabling cells to respond to mechanical signals [166]. The stiffness and migration ability of cancer cells change in response to matrix stiffening [167]. Tumor cells gradually soften during metastasis, and low cell stiffness is associated with high metastatic potential. Increased cell stiffness impairs cell motility, whereas decreased stiffness promotes cell migration and invasion [168]. Cancer cells attenuate their motility by activating the JNK signaling pathway [169]. Furthermore, the mechanical properties of tumor cells determine their self‐renewal through cytoskeletal and Wnt/β‐catenin signaling [170]. Upregulation of Ras/MAPK signaling is accompanied by actin cytoskeletal restructuring and cell softening [171]. Cancer progression is promoted by these mechanical changes, which facilitate cellular motility in physically confined settings [172].

The Hippo signaling pathway is closely associated with changes in mechanical forces and ECM adhesion [173]. YAP is an effector of the Hippo signaling pathway and a key mediator of mechanotransduction. When cells grow on a stiff matrix, YAP is primarily localized in the nucleus. Moreover, mechanical signals can induce the nucleocytoplasmic translocation of YAP by modulating the structure of nuclear pores [174, 175, 176]. Changes in mechanical forces can induce microfilament depolymerization, which in turn regulates YAP distribution, and this exerts a more pronounced effect on tumor cell proliferation and migration [177]. Disheveled‐associated activator of morphogenesis 1 enhances mechanotransduction by activating the RhoA/YAP signaling axis, thereby promoting microfilament remodeling and increasing the migratory ability of tumor cells [178]. In Grade III breast carcinomas, the level of YAP gene signature is higher and correlates with tumor stiffness [179].

Several cytoskeletal proteins and their modulators have been implicated in stiffness‑regulated metastasis and tumor progression. An actin‐binding protein‐modified magnetic nanomotor coupled with a rotating magnetic field can target the actin cytoskeleton and induce its depolymerization, leading to reduced matrix stiffness of tumor cells and suppressed tumor growth [180, 181]. Kinesin family member 20A (KIF20A) overexpression in bladder cancer is linked to cortical stiffness changes via its interaction with Myosin IIA, and its inhibition reduces cell motility [182]. PDAC cell stiffness, meanwhile, is determined by the combined activities of Myosin II, Arp2/3, and formins [183]. Functionally, actomyosin blockade impairs invasion in single and collective cells, whereas actin perturbation may provoke a metastasis‑enhancing escape behavior in individual cancer cells [184]. The actin‐crosslinking protein palladin promotes matrix stiffness by regulating force generation and mechanosensitivity of cancer‐associated fibroblasts [185]. S‐nitrosylation of ezrin promotes the metastasis of NSCLC cells by facilitating mechanotransduction [186].

Nestin, a member of the Type VI intermediate filament family, is abundantly expressed in various aggressively metastatic tumor cells, reflecting the broader role of intermediate filaments in governing cell stiffness and invasive behavior. Expression of nestin increases the mobility of vimentin filaments, leading to reduced cell stiffness [187]. Moreover, nestin can absorb mechanical load and maintain cytoskeletal integrity [188].

### 4.2 Stress

Activated by shear stress, cellular signaling contributes to the control of migration and proliferation. Shear stress reduces the stiffness of cancer cells via the FAK–ERK1/2 signaling pathway, thereby promoting liver cancer migration [189]. Shear stress induces adaptations in the cell membrane and actin cytoskeleton, while regulating the nuclear translocation of sterol regulatory element‐binding protein 2 (SREBP2) and YAP1, thereby promoting increased chemoresistance [190]. Elevated solid stress in solid tumors is closely associated with the expression of CKAP4. CKAP4 binds to microtubules and subsequently remodels them, thereby enhancing cell spreading and promoting distant metastasis in vivo [191]. Furthermore, intermediate filaments support leader cell invasion by buffering nuclear deformation under compressive stress and by mediating mechanosensitive matrix degradation [192]. Through cortical tension, actomyosin contractility not only drives cell shape changes during mitosis but also links to the maintenance of mitotic spindle integrity [193].

Taken together, the mechanical attributes of cells are markedly affected by cytoskeletal changes, which in turn are tightly associated with the growth and invasive potential of malignant cells [194, 195].

## 5 Cytoskeletal Dynamics in Cancer Therapy

**FIGURE 5:**
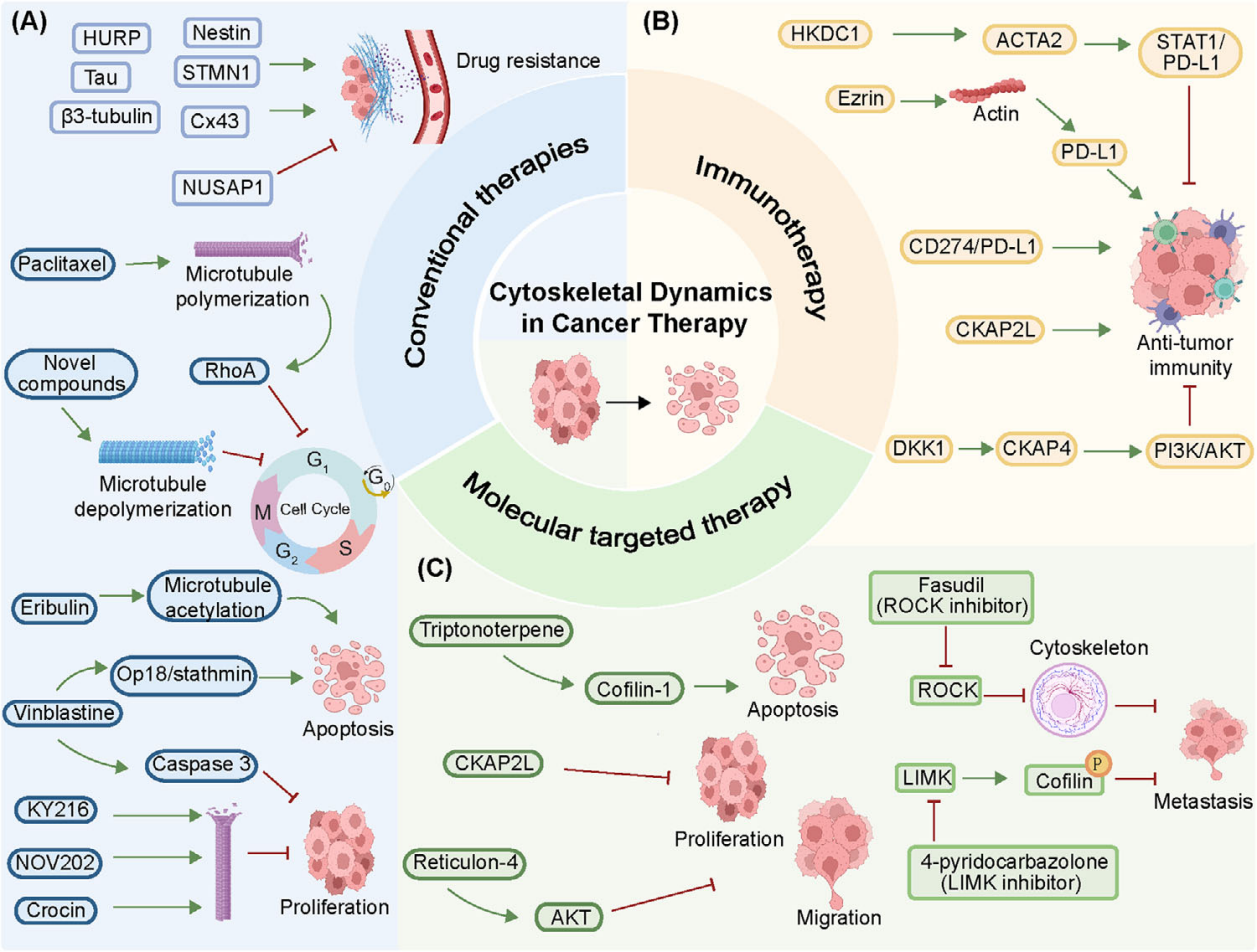
Cytoskeletal dynamics in cancer therapy. (A) Conventional therapies. HURP, NUSAP1, Tau, β3‑tubulin, nestin, STMN1, and Cx43 are aberrantly expressed in cancers and modulate chemotherapy response. Paclitaxel polymerizes microtubules, regulates RhoA, and induces cell cycle arrest. Vinblastine promotes apoptosis via Op18/stathmin, activates Caspase‑3, and inhibits proliferation. Eribulin triggers microtubule acetylation, leading to apoptosis. Microtubule inhibitors (KY216, NOV202, and crocin) and numerous novel compounds selectively target microtubules to suppress cancer cell proliferation and metastasis, exhibiting potent antitumor activity. (B) Immunotherapy. HKDC1 binds ACTA2 to activate STAT1/PD‑L1 signaling, promoting immune escape. Ezrin bridges membrane to actin and positively regulates PD‑L1 expression. DKK1 interacts with macrophage CKAP4 to activate PI3K–AKT, suppressing NK/CD8^+^ T cell responses; blocking this interaction impairs actin remodeling. CKAP2L correlates with immune subtypes and infiltration, serving as a potential predictor of immunotherapy responsiveness. (C) Molecular targeted therapy. Triptonoterpene regulates actin dynamics and promotes Cofilin‑1 mitochondrial translocation to induce apoptosis. CKAP2L knockdown suppresses proliferation and metastasis while inducing G2/M arrest. Reticulon‑4 knockdown destabilizes tubulin and enhances chemosensitivity via AKT downregulation. Fasudil (ROCK inhibitor) suppresses ovarian cancer invasion by blocking ROCK activation and disrupting cytoskeletal remodeling. 4‑Pyridocarbazolone (LIMK inhibitor) inhibits LIMK‑mediated cofilin phosphorylation, and effectively suppresses tumor metastasis.

Cytoskeletal dynamics are gaining increasing attention in cancer therapy (Figure 5). They influence the response of tumor cells through various mechanisms, including conventional therapy, immunotherapy, and molecular targeted therapy.

### 5.1 Conventional Therapies

Conventional cancer treatments include chemotherapy and radiotherapy. However, tumor cells often develop resistance to these therapies through various mechanisms. Cytoskeletal dynamics play a crucial role in regulating tumor resistance, with the majority of evidence focusing on microtubule‑associated proteins and their impact on chemotherapeutic efficacy (Figure 5A).

Chemotherapy resistance remains a major obstacle in cancer treatment. Several MAPs have been implicated in this process. HURP is upregulated in various cancer types and enhances drug resistance. Cryo‑electron microscopy analysis revealed an interaction between the tubulin‑binding domain of HURP and the vinca domain on β‑tubulin, which is the target site of Vinca alkaloids [196]. NUSAP1 is significantly upregulated in lung adenocarcinoma and is markedly associated with increased sensitivity to chemotherapeutic agents, contrasting with the resistance‑promoting role of other proteins [197]. Tau is associated with increased drug resistance in various cancers. Prostate cancer cells that relapse after docetaxel treatment exhibit higher levels of the MAPT [198]. Overexpression of β3‑tubulin is similarly linked to resistance to MTA [199, 200]. In melanoma, nestin depletion affects signaling through the integrin and PI3K/AKT/mTOR pathways, leading to increased abundance and phosphorylation of FA kinase, which is associated with acquired resistance to vemurafenib [201]. High expression of STMN1 is correlated with poor response to docetaxel‑based chemotherapy [202]. Beyond these individual proteins, the increased direct interaction between the gap junction protein connexin43 (Cx43) and microtubules plays an important role in chemotherapy resistance in glioblastoma. Disrupting this interaction in glioma stem cells significantly enhances the efficacy of chemotherapy [203]. Furthermore, STAT3 is activated in paclitaxel‑resistant cells, whereas STMN is inactivated. STAT3 inhibitors reverse paclitaxel resistance by inhibiting the STAT3–STMN interaction, thereby promoting cell death [204].

Microtubules are a well‐established target for anticancer pharmacotherapy, with examples including paclitaxel, vinblastine, and eribulin. Paclitaxel‐induced microtubule polymerization in cancer cells regulates the level of RhoA and induces cell cycle arrest [205]. Vinblastine promotes apoptosis in cancer cells via Op18/STMN, activates Caspase 3, and inhibits cell proliferation and migration [206]. Microtubule acetylation and PERK activation elicited by eribulin give rise to endoplasmic reticulum‑to‑mitochondria calcium overload, with subsequent apoptotic cell death [207].

MTAs primarily kill cancer cells during mitosis. Microtubule inhibitors, such as KY216, NOV202, and crocin, bind to microtubule to inhibit cancer cell proliferation and metastasis, demonstrating potent anticancer efficacy [208, 209, 210]. However, mitotic death can be circumvented by a subset of cancer cells following MTA treatment, which then exit mitosis and give rise to polyploid giant cancer cells (PGCCs). ST‐401, a mild microtubule assembly inhibitor that preferentially kills cancer cells during interphase, can avoid the development of PGCCs [211].

In recent years, many novel compounds have been developed that selectively target microtubule to exert antitumor effects. Among them, 4‐(6‐((3‐methoxyphenyl)amino)pyrimidin‐4‐yl)‐N,N‐dimethylbenzenamine selectively targets gamma‐tubulin 1 to inhibit the progression of lung cancer [212]. A novel selenium‐containing lead compound 2 g binds to the colchicine site of tubulin, leading to G2/M cell cycle arrest [213]. Erianin derivatives can inhibit microtubule polymerization, induce intracellular ferroptosis, and exhibit excellent antitumor activity in NSCLC [214]. Silybin derivatives inhibit tubulin activity and the expression of EMT‐related proteins, while upregulating the antimetastatic protein Cyclin B1, thereby suppressing CRC metastasis [215]. Furthermore, some novel compounds, such as 1,2,3‐triazole arylamide derivatives, tetrazolo[1,5‐a]pyrimidine, thienopyridine indole derivatives, and nicotinic acid derivatives, promote microtubule depolymerization, induce G2/M phase arrest, and facilitate apoptosis in cancer cells [216, 217, 218, 219, 220, 221, 222].

### 5.2 Immunotherapy

Immunotherapy represents a major advancement in cancer treatment (Figure 5B). However, many solid tumors, particularly the immunosuppressive tumor microenvironment they create, suppress the expression of innate immune molecules, thereby limiting immune cell infiltration. Actin cytoskeleton remodeling drives cancer cells to evade NK cell‐mediated cytotoxicity [223]. Furthermore, cytoskeletal dynamics influence the immune response within the TME by regulating immune cell infiltration.

Immune checkpoint blockade (ICB) therapy shows great promise in treating various malignant tumors. However, immune escape severely weakens the therapeutic effect of ICB. Hexokinase domain component 1 (HKDC1) activates the STAT1/PD‐L1 signaling pathway in tumor cells by binding to the cytoskeletal protein ACTA2 [224]. This ultimately promotes tumor immune escape. Immune checkpoint inhibitors (ICIs) targeting CD274/PD‐L1 have demonstrated significant clinical efficacy. As an immune checkpoint molecule, PD‑L1 is localized to the plasma membrane of numerous cancer cells and serves to suppress T‑cell‑mediated immune monitoring. Ezrin is a crucial actin‑binding protein that bridges the plasma membrane to actin filaments during lamellipodia formation, cell polarization, and migration [225]. Furthermore, interfering with ezrin expression significantly reduces the expression of PD‐L1 [226].

Tumor‑associated macrophages are critical for driving immune evasion, thus compromising the efficacy of ICIs in cancer therapy. By interacting with CKAP4 on macrophages and activating PI3K–AKT signaling, dickkopf‐1 (DKK1) suppresses the antitumor immune responses mediated by NK cells and CD8^+^ T cells [227]. A recent study designed an aptamer inhibitor that blocks the interaction between CKAP4 and DKK1, thereby inhibiting the PI3K/AKT signaling pathway and consequently impairing the reorganization of the actin cytoskeleton during cell migration [228]. Therefore, DKK1 represents a potential target for enhancing PD‐1 blockade therapy in cancer. Furthermore, the association of CKAP2L with immune subtypes, immune cell infiltration, and the tumor immune landscape highlights its promise as a predictor of responsiveness to cancer immunotherapy [81].

Microtubule inhibitors can also enhance the efficacy of immunotherapy by disrupting microtubule dynamics. Combretastatin A4, a microtubule inhibitor that targets tumor vascular endothelial cells, effectively inhibits microtubules in tumor endothelial cells and enhances T cell infiltration and activation [229]. Therefore, targeting microtubules represents a promising immunomodulatory strategy.

### 5.3 Molecular Targeted Therapy

Molecular targeted therapy provides effective treatment by targeting key molecules in tumor cells (Figure 5C). Cytoskeletal dynamics play an important role in this therapy. Katanin is a critical cytoskeletal component that plays a major role in microtubule severing. To date, a compound, PubChem CID 122589735, has been identified that inhibits katanin, representing a novel anticancer therapy [230]. Triptonoterpene exerts proapoptotic effects on gastric cancer cells by virtue of its regulation of actin dynamics and its promotion of Cofilin‑1 translocation to mitochondria [231]. The actin cytoskeleton is specifically targeted and disrupted by core‑shell metal‑organic framework nanoparticles, resulting in inhibition of cancer cell migration [232]. Knockdown of CKAP2L markedly suppresses the proliferation and metastatic capacity of KIRC cells while triggering G2/M cell‑cycle arrest [81]. Knockdown of reticulon‐4 affects tubulin stability and enhances the cytotoxic effect of chemotherapeutic drugs on cancer cells by downregulating the AKT signaling pathway [233].

Targeting regulators of the cytoskeleton can also provide effective therapy. ROCK is a regulator of the actin cytoskeleton and is frequently overexpressed in various malignant tumors. ROCK inhibitors possess strong anticancer activity and promote apoptosis [234]. Novel pyrazolo[1,5‐a]pyrimidine derivatives act as ROCK inhibitors and suppress cancer metastasis [235]. LIMK2 plays a critical role in regulating actin cytoskeleton and influences cancer cell proliferation. ChemDiv‐8020‐2508, NCI300395 and ChemDiv‐7997‐0024 are potential candidates for cancer therapies targeting LIMK2 [236]. Tau is a promoter of tubulin assembly into microtubules, and its dysregulated expression is associated with various cancers [237, 238]. Flavonoid hybrid derivatives target the Tau protein and exert anticancer effects in glioblastoma [237].

Taken together, these findings demonstrate that cytoskeletal dynamics, particularly those involving microtubules and their associated proteins, are central to both the efficacy and resistance of cancer therapies. The development of novel microtubule‑targeting compounds offers promising strategies to overcome resistance. Continued elucidation of these mechanisms will be essential for the design of anticancer agents.

## 6 Cytoskeletal Dynamics in Cancer Clinical Applications

Targeting the cytoskeleton has emerged as a novel therapeutic approach against chemotherapy‑resistant tumors, with a growing body of clinical research in this area, offering encouraging prospects for cancer treatment. Cytoskeletal dynamics related to cancer clinical applications are summarized in Table 4.

**TABLE 4:** Clinical trials of cytoskeleton targeting chemotherapeutic agent.

NCT number	Cytoskeleton type	Drug/treatment	Cancer type	Status
NCT00836160	Microtubule	The third‐line antimicrotubule agents	Non‐small‐cell lung cancer	Completed
NCT03393741	Microtubule	Antimicrotubule drugs (taxane, eribulin, vinorelbine, and ixabepilone)	Breast cancer	Terminated
NCT02171260	Microtubule	Eribulin mesylate	Recurrent or refractory solid tumors	Completed
NCT03250299	Microtubule	BAL101553	Glioblastoma	Terminated
NCT01469598	Microtubule	Docetaxel	Esophageal squamous cell cancer	Completed
NCT04823897	Microtubule	CCI‐001	Recurrent and/​or metastatic solid tumors	Recruiting
NCT00423410	Microtubule	EPC2407	Solid tumor or lymphoma	Completed
NCT07439094	Microtubule	CKD‐703	Non‐small‐cell lung cancer	Recruiting
NCT04503265	Microtubule	AMXI‐5001	Advanced malignancies	Recruiting
NCT02097238	Microtubule	Eribulin mesylate	Recurrent or refractory osteosarcoma	Completed
NCT03482362	Microtubule	Vinorelbine tartrate	Advanced BRAF‐like colon cancer	Completed
NCT04683445	Microtubule	Eribulin	Advanced breast cancer	Unknown status
NCT01186731	Microtubule	Liposome‐entrapped docetaxel	Locally advanced or metastatic pancreatic cancer	Completed
NCT06548672	Microtubule	BC3195	Advanced or metastatic cancer	Recruiting
NCT03257891	Microtubule	Cabazitaxel	Adrenocortical Carcinoma	Unknown status
NCT04662580	Microtubule	ARX517	Metastatic prostate cancer	Active, not recruiting
NCT02404506	Microtubule	Eribulin mesilate	Advanced breast cancer	Terminated
NCT06857305	Microtubule	Eribulin biweekly regimen Eribulin standard regimen	Locally recurrent or metastatic HER2‐negative breast cancer	Enrolling by invitation
NCT01913652	Microtubule	Cabazitaxel	Dedifferentiated liposarcoma	Completed
NCT02037529	Microtubule	Eribulin mesylate, paclitaxel	Recurrent Stage IIIC–IV breast cancer	Suspended
NCT02047214	Microtubule	TPI 287	Glioblastoma multiforme	Terminated

*Note*: Data were acquired from NIH‐ClinicalTrials.gov (https://clinicaltrials.gov/).

### 6.1 Actin Filament‐Targeting Agents

Actin filament‑targeting drugs exert antimetastatic effects by disrupting actin dynamics, thereby inhibiting the invasive capacity of cancer cells. Although this target has attracted considerable attention, the presence of actin filaments in normal cells often leads to severe adverse effects associated with such agents. Consequently, their development remains confined to the preclinical stage [239], and to date, no actin filament‑targeting drug has received approval from the United States Food and Drug Administration (US FDA).

Given that direct targeting of actin filaments frequently results in normal tissue toxicity, research interest has now increasingly focused on upstream regulatory molecules of actin filaments, including kinases such as ROCK and LIMK. ROCK serves as a core component of the Rho signaling pathway and exerts a critical function in the metastatic process of cancer. Fasudil, a ROCK inhibitor approved for clinical use in China, effectively suppresses ovarian cancer cell invasion by blocking ROCK activation and disrupting cytoskeletal remodeling [240]. LIMK serves as a critical downstream kinase in the ROCK signaling pathway, stabilizing actin filaments through the phosphorylation and inactivation of cofilin. The compound 4‑pyridocarbazolone, a selective LIMK inhibitor, acts by blocking LIMK‑mediated cofilin phosphorylation, which in turn promotes actin depolymerization and reduces the formation of cellular invasive protrusions, consequently exerting significant suppression on tumor cell proliferation and metastatic potential [241]. Since ROCK and LIMK inhibitors are capable of disrupting cytoskeletal remodeling, targeting these upstream regulatory molecules may offer a viable strategy for cytoskeleton‑based therapy.

### 6.2 Microtubule‑Targeting Agents

With regard to clinical research on the cytoskeleton in cancer, the overwhelming majority of efforts are currently directed toward microtubules, and to date, only MTA have been granted marketing authorization by US FDA. Microtubules are involved in spindle assembly and are indispensable for rapidly proliferating cancer cells, thereby constituting highly attractive targets for the development of anticancer therapeutics. MTAs are broadly categorized into two classes: microtubule‑stabilizing agents and microtubule‑destabilizing agents. Microtubule‑stabilizing agents act by promoting the polymerization of tubulin into microtubules and preventing their depolymerization, thereby inducing aberrant microtubule stabilization and impairing normal spindle function. Taxanes represent the classic microtubule‑stabilizing agents, with representative drugs including paclitaxel, docetaxel, and cabazitaxel, which are clinically employed in the management of lung, breast, prostate, and other malignancies [242, 243, 244, 245]. Microtubule‑destabilizing agents are capable of suppressing tubulin polymerization or accelerating the disassembly of preformed microtubules. Vinca alkaloids serve as classic microtubule‑destabilizing anticancer agents, with vinblastine and vincristine being representative examples [246, 247]. Their mechanism of action involves binding to β‑tubulin, perturbing microtubule dynamics, and subsequently inducing apoptosis. In clinical practice, these two drugs are primarily indicated for the treatment of lymphoma and breast cancer [248, 249]. All the above‑mentioned MTAs have been granted marketing authorization by US FDA.

Nevertheless, prolonged administration of MTAs is often associated with acquired resistance. In this context, P‑glycoprotein functions as a drug efflux pump that actively transports taxanes out of tumor cells, ultimately conferring resistance to taxanes [250]. Cabazitaxel is a semi‑synthetic analog of docetaxel that was developed as a strategy to overcome drug resistance. It exhibits low affinity for P‑glycoprotein, thereby retaining antitumor activity against drug‑resistant cancer cells. Cabazitaxel was approved by the US FDA in 2010 for the treatment of metastatic prostate cancer patients whose disease had progressed on docetaxel [251]. Furthermore, additional clinical studies have demonstrated that cabazitaxel is also highly effective in patients with metastatic cancer, even in those who are resistant to taxane‑based agents [252]. Furthermore, Ixabepilone acts by stabilizing microtubules, thereby inhibiting mitosis. Owing to its low affinity for P‑glycoprotein, this agent retains its antitumor activity even in tumor cells that have acquired resistance to conventional MTAs. Ixabepilone was approved by the US FDA in 2007 for patients with advanced metastatic cancer who had progressed after taxane‑based chemotherapy [253].

### 6.3 Intermediate Filament‐Targeting Agents

Intermediate filaments constitute a principal component of the cytoskeleton and display distinct tissue‑specific expression patterns. Nevertheless, given their involvement in normal physiological processes, including immune responses and tissue repair, their inhibition is likely to compromise the function of normal tissues [254]. To date, therefore, no anticancer agent targeting intermediate filaments has advanced into clinical investigation, nor has the US FDA granted approval to any such agent.

Taken together, although cytoskeleton‑targeting agents have shown remarkable efficacy in clinical cancer treatment, their widespread use is severely constrained by normal cell toxicity. Therefore, alleviating adverse effects on healthy tissues represents a critical challenge that must be addressed in the current development of these drugs. Future investigations should prioritize the design of precision delivery systems that enable targeted therapy toward malignant cells, thereby improving the therapeutic window and reducing systemic toxicity.

## 7 Conclusion and Future Perspectives

This study explores the changes in the cytoskeleton in cancer, as well as its upstream and downstream regulatory mechanisms. Additionally, it introduces the mechanical microenvironment closely associated with the cytoskeleton and how it shapes tumor progression. The cytoskeleton is involved in multiple aspects of cancer cell proliferation, including metabolic reprogramming, differential protein expression, signaling pathway regulation, and cell cycle control. Furthermore, it participates in tumor metastasis, cell death, and angiogenesis, thereby providing a foundation for tumor development. A key mechanism of cancer metastasis is EMT, which enhances the migratory ability of cancer cells by converting epithelial cells into mesenchymal cells, thereby promoting tumor metastasis. The cytoskeleton regulates EMT‐related signaling pathways and influences cancer metastasis. Moreover, the cytoskeleton plays a critical role in programmed cell death, including apoptosis, autophagy, and disulfidptosis.

Therapeutic approaches targeting cytoskeletal dynamics have rapidly emerged as a promising direction in cancer treatment. Conventional strategies such as chemotherapy, radiotherapy, and some targeted therapies primarily aim to induce apoptosis. However, resistance to apoptosis, particularly in advanced or refractory tumors, remains a major obstacle. To address this issue, various microtubule‐targeting compounds have been developed, which inhibit microtubule dynamics and reverse chemotherapy resistance. Furthermore, the cytoskeleton regulates the infiltration and activation of immune cells, thereby influencing cancer immunotherapy; it also mediates the expression of immune checkpoints, promoting tumor immune evasion.

The cytoskeleton holds great promise as a therapeutic target in oncology. MTAs have demonstrated notable efficacy in clinical cancer treatment. However, their application is severely constrained by off‑target toxicity to normal cells. Consequently, mitigating adverse effects on healthy tissues represents a critical challenge that must be addressed in current drug development efforts. Future research should prioritize the design of precision delivery systems capable of specifically targeting malignant cells, thereby improving the therapeutic window and reducing systemic toxicity. In parallel, the development of inhibitors directed against other cytoskeletal components may offer novel and more selective therapeutic options. Advancing our understanding of cytoskeleton will facilitate the design of more effective treatment regimens, ultimately improving patient survival outcomes and quality of life.

## Author Contributions

**Jie Chen**: writing – original draft, visualization, and formal analysis. **Wenxi Yang**: writing – original draft. **Yonghan Song**: writing – original draft. **Yuanhao Zhang**: writing – review and editing and Visualization. **Jia Ma**: writing – review and editing, visualization, and data curation. **Tingyao Wang**: visualization and data curation. **Jijun Zheng**: visualization and data curation. **Hebin Zhang**: visualization. **Yiyao Liu**: writing – review and editing and conceptualization. **Jinhao Zeng**: writing – review and editing and conceptualization. All authors have read and approved the final manuscript.

## Ethics Statement

The authors have nothing to report.

## Conflicts of Interest

The authors declare no conflicts of interest.

## Data Availability

The authors have nothing to report.

## References

[1] D. Hanahan and R. A. Weinberg , “Hallmarks of Cancer: the next Generation,” Cell 144, no. 5 (2011): 646–674. 10.1016/j.cell.2011.02.013 21376230

[2] N. M. Anderson and M. C. Simon , “The Tumor Microenvironment,” Current Biology: CB 30, no. 16 (2020): R921–R925. 10.1016/j.cub.2020.06.081 PMC8194051 32810447

[3] F. Bray , M. Laversanne , H. Sung , et al., “Global Cancer Statistics 2022: GLOBOCAN Estimates of Incidence and Mortality Worldwide for 36 Cancers in 185 Countries,” CA: a Cancer Journal for Clinicians 74, no. 3 (2024): 229–263. 10.3322/caac.21834 38572751

[4] S. Zhang , X. Xiao , Y. Yi , et al., “Tumor Initiation and Early Tumorigenesis: Molecular Mechanisms and Interventional Targets,” Signal Transduction and Targeted Therapy 9, no. 1 (2024): 149. 10.1038/s41392-024-01848-7 PMC11189549 38890350

[5] M. Greaves and C. C. Maley , “Clonal Evolution in Cancer,” Nature 481, no. 7381 (2012): 306–313. 10.1038/nature10762 PMC3367003 22258609

[6] E. Piskounova , M. Agathocleous , M. M. Murphy , et al., “Oxidative Stress Inhibits Distant Metastasis by human Melanoma Cells,” Nature 527, no. 7577 (2015): 186–191. 10.1038/nature15726 PMC4644103 26466563

[7] M. Li , M. Hu , L. Jiang , J. Pei , and C. Zhu , “Trends in Cancer Incidence and Potential Associated Factors in China,” JAMA Network Open 7, no. 10 (2024): e2440381. 10.1001/jamanetworkopen.2024.40381 PMC11581522 39432306

[8] C. de Martel , D. Georges , and F. Bray , “Global Burden of Cancer Attributable to Infections in 2018: a Worldwide Incidence Analysis,” The Lancet Global Health 8, no. 2 (2020): e180–e190. 10.1016/S2214-109X(19)30488-7 31862245

[9] N. Y. Nair , V. Samuel , L. Ramesh , et al., “Actin Cytoskeleton in Angiogenesis,” Biology Open 11, no. 12 (2022): bio058899. 10.1242/bio.058899 PMC9729668 36444960

[10] T. Kundu , P. Dutta , D. Nagar , S. Maiti , and A. Ghose , “Coupling of Dynamic Microtubules to F‐actin by Fmn2 Regulates Chemotaxis of Neuronal Growth Cones,” Journal of Cell Science 134, no. 13 (2021): jcs252916. 10.1242/jcs.252916 34313311

[11] M. Wu , C. Sarkar , and B. Guo , “Regulation of Cancer Metastasis by PAK2,” International Journal of Molecular Sciences 25, no. 24 (2024): 13443. 10.3390/ijms252413443 PMC11676821 39769207

[12] M. E. Wawro , K. Chojnacka , K. Wieczorek‐Szukala , K. Sobierajska , and J. Niewiarowska , “Invasive Colon Cancer Cells Induce Transdifferentiation of Endothelium to Cancer‐Associated Fibroblasts through Microtubules Enriched in Tubulin‐β3,” International Journal of Molecular Sciences 20, no. 1 (2018): 53. 10.3390/ijms20010053 PMC6337286 30583584

[13] N. A. Kuburich , P. den Hollander , J. T. Pietz , and S. A. Mani , “Vimentin and Cytokeratin: Good Alone, Bad Together,” Seminars in Cancer Biology 86, no. Pt 3 (2022): 816–826. 10.1016/j.semcancer.2021.12.006 PMC9213573 34953942

[14] D. A. Fletcher and R. D. Mullins , “Cell Mechanics and the Cytoskeleton,” Nature 463, no. 7280 (2010): 485–492. 10.1038/nature08908 PMC2851742 20110992

[15] A. Mannucci and A. Goel , “Advances in Pancreatic Cancer Early Diagnosis, Prevention, and Treatment: the Past, the Present, and the Future,” CA: A Cancer Journal for Clinicians 76, no. 1 (2026): e70035. 10.3322/caac.70035 PMC12788368 40971231

[16] K. E. de Visser and J. A. Joyce , “The Evolving Tumor Microenvironment: from Cancer Initiation to Metastatic Outgrowth,” Cancer Cell 41, no. 3 (2023): 374–403. 10.1016/j.ccell.2023.02.016 36917948

[17] S. Lim , S. Woo , K. W. Lee , and K. D. Kim , “Roles of Cytoskeleton in Metastasis: from Its Mechanism to Therapeutic Strategies,” Experimental & Molecular Medicine 58, no. 1 (2026): 1–13. 10.1038/s12276-025-01608-9 PMC12868002 41495421

[18] L. Mosca , A. Ilari , F. Fazi , Y. G. Assaraf , and G. Colotti , “Taxanes in Cancer Treatment: Activity, Chemoresistance and Its Overcoming,” Drug Resistance Updates 54 (2021): 100742. 10.1016/j.drup.2020.100742 33429249

[19] C. Zhang , M. Zhao , G. Wang , and Y. Li , “Recent Progress on Microtubule Degradation Agents,” Journal of Medicinal Chemistry 66, no. 19 (2023): 13354–13368. 10.1021/acs.jmedchem.3c00517 37748178

[20] S. Seetharaman and S. Etienne‐Manneville , “Cytoskeletal Crosstalk in Cell Migration,” Trends in Cell Biology 30, no. 9 (2020): 720–735. 10.1016/j.tcb.2020.06.004 32674938

[21] T. Hohmann and F. Dehghani , “The Cytoskeleton‐A Complex Interacting Meshwork,” Cells 8, no. 4 (2019): 362. 10.3390/cells8040362 PMC6523135 31003495

[22] W. Oosterheert , M. Boiero Sanders , J. Funk , D. Prumbaum , S. Raunser , and P. Bieling , “Molecular Mechanism of Actin Filament Elongation by Formins,” Science (New York, NY) 384, no. 6692 (2024): eadn9560. 10.1126/science.adn9560 38603491

[23] Y. H. Tee , W. J. Goh , X. Yong , et al., “Actin Polymerisation and Crosslinking Drive Left‐right Asymmetry in Single Cell and Cell Collectives,” Nature Communications 14, no. 1 (2023): 776. 10.1038/s41467-023-35918-1 PMC9922260 36774346

[24] R. Socodato and J. B. Relvas , “A Cytoskeleton Symphony: Actin and Microtubules in Microglia Dynamics and Aging,” Progress in Neurobiology 234 (2024): 102586. 10.1016/j.pneurobio.2024.102586 38369000

[25] A. Valdivia , C. Duran , M. Lee , H. C. Williams , M.‐Y. Lee , and A. San Martin , “Nox1‐based NADPH Oxidase Regulates the Par Protein Complex Activity to Control Cell Polarization,” Frontiers in Cell and Developmental Biology 11 (2023): 1231489. 10.3389/fcell.2023.1231489 PMC10457011 37635877

[26] C. Ruggiero and E. Lalli , “Targeting the Cytoskeleton against Metastatic Dissemination,” Cancer and Metastasis Reviews 40, no. 1 (2021): 89–140. 10.1007/s10555-020-09936-0 33471283

[27] J. Chikireddy , L. Lengagne , R. Le Borgne , et al., “Fascin‐induced Bundling Protects Actin filaments from Disassembly by Cofilin,” Journal of Cell Biology 223, no. 6 (2024): e202312106. 10.1083/jcb.202312106 PMC10949937 38497788

[28] N. Koundinya , R. M. Aguilar , K. Wetzel , et al., “Two Ligands of Arp2/3 Complex, Yeast Coronin and GMF, Interact and Synergize in Pruning Branched Actin Networks,” Journal of Biological Chemistry 301, no. 3 (2025): 108191. 10.1016/j.jbc.2025.108191 PMC11872438 39826693

[29] D. Londoño‐Vásquez , K. Rodriguez‐Lukey , S. K. Behura , et al., “Microtubule Organizing Centers Regulate Spindle Positioning in Mouse Oocytes,” Developmental Cell 57, no. 2 (2022): 197–211. 10.1016/j.devcel.2021.12.011 PMC8792338 35030327

[30] J. Krüger , A. Fischer , M. Breunig , et al., “DNA Methylation‐associated Allelic Inactivation Regulates Keratin 19 Gene Expression during Pancreatic Development and Carcinogenesis,” The Journal of Pathology 261, no. 2 (2023): 139–155. 10.1002/path.6156 37555362

[31] L. Pradeau‐Phélut and S. Etienne‐Manneville , “Cytoskeletal Crosstalk: a Focus on Intermediate filaments,” Current Opinion in Cell Biology 87 (2024): 102325. 10.1016/j.ceb.2024.102325 38359728

[32] X. Huang , S. Zhao , Y. Xing , et al., “The Unconventional Role of vimentin Intermediate filaments,” Current Opinion in Cell Biology 93 (2025): 102483. 10.1016/j.ceb.2025.102483 39978207

[33] M. Bates , J. Keller‐Findeisen , A. Przybylski , et al., “Optimal Precision and Accuracy in 4Pi‐STORM Using Dynamic Spline PSF Models,” Nature Methods 19, no. 5 (2022): 603–612. 10.1038/s41592-022-01465-8 PMC9119851 35577958

[34] A. Bharadwaj , A. Kumar , S. Padalumavunkal Mathew , et al., “Advancing Cellular Insights: Super‐resolution STORM Imaging of Cytoskeletal Structures in human Stem and Cancer Cells,” Biochemistry and Biophysics Reports 39 (2024): 101798. 10.1016/j.bbrep.2024.101798 PMC11332080 39161577

[35] C. Bond , A. N. Santiago‐Ruiz , Q. Tang , and M. Lakadamyali , “Technological Advances in Super‐resolution Microscopy to Study Cellular Processes,” Molecular Cell 82, no. 2 (2022): 315–332. 10.1016/j.molcel.2021.12.022 PMC8852216 35063099

[36] Y. Mo , K. Wang , L. Li , et al., “Quantitative Structured Illumination Microscopy via a Physical Model‐based Background Filtering Algorithm Reveals Actin Dynamics,” Nature Communications 14, no. 1 (2023): 3089. 10.1038/s41467-023-38808-8 PMC10227022 37248215

[37] C. Qiao , D. Li , Y. Guo , et al., “Evaluation and Development of Deep Neural Networks for Image Super‐resolution in Optical Microscopy,” Nature Methods 18, no. 2 (2021): 194–202. 10.1038/s41592-020-01048-5 33479522

[38] J. Park , Y. Wu , J. Suk Kim , J. Byun , J. Lee , and Y.‐K. Oh , “Cytoskeleton‐modulating Nanomaterials and Their Therapeutic Potentials,” Advanced Drug Delivery Reviews 211 (2024): 115362. 10.1016/j.addr.2024.115362 38906478

[39] X. Ni , C.‐P. Lu , G.‐Q. Xu , and J.‐J. Ma , “Transcriptional Regulation and Post‐translational Modifications in the Glycolytic Pathway for Targeted Cancer Therapy,” Acta Pharmacologica Sinica 45, no. 8 (2024): 1533–1555. 10.1038/s41401-024-01264-1 PMC11272797 38622288

[40] D. Dutta , M. Ziemke , P. Sindelar , H. Vargas , J. Y. Lim , and S. Chandra , “Cytoarchitecture of Breast Cancer Cells under Diabetic Conditions: Role of Regulatory Kinases—Rho Kinase and Focal Adhesion Kinase,” Cancers 16, no. 18 (2024): 3166. 10.3390/cancers16183166 PMC11430325 39335137

[41] Y. Yang , Y. Wang , H. Qu , et al., “CircCDYL Promotes Glycolysis to Drive the Progression of Nasopharyngeal Carcinoma,” Journal of Advanced Research 84 (2025): 445–459. 10.1016/j.jare.2025.09.053 PMC13227178 41016592

[42] Q. Wang , M. Li , C. Chen , et al., “Glucose Homeostasis Controls N‐acetyltransferase 10‐mediated ac4C Modification of HK2 to Drive Gastric Tumorigenesis,” Theranostics 15, no. 6 (2025): 2428–2450. 10.7150/thno.104310 PMC11840738 39990211

[43] D. Che , M. Wang , J. Sun , et al., “KRT6A Promotes Lung Cancer Cell Growth and Invasion through MYC‐Regulated Pentose Phosphate Pathway,” Frontiers in Cell and Developmental Biology 9 (2021): 694071. 10.3389/fcell.2021.694071 PMC8255478 34235156

[44] H. Li , C. Song , Y. Zhang , et al., “Transgelin Promotes Glioblastoma Stem Cell Hypoxic Responses and Maintenance through p53 Acetylation,” Advanced Science (Weinheim, Baden‐Wurttemberg, Germany) 11, no. 7 (2024): e2305620. 10.1002/advs.202305620 PMC10870072 38087889

[45] R. Cornelison , L. Marrah , A. Fierti , et al., “The Potential for Targeting AVIL and Other Actin‐Binding Proteins in Rhabdomyosarcoma,” International Journal of Molecular Sciences 24, no. 18 (2023): 14196. 10.3390/ijms241814196 PMC10531751 37762498

[46] N. Y. Yeat , L.‐H. Liu , Y.‐H. Chang , C. P.‐K. Lai , and R.‐H. Chen , “Bro1 proteins Determine Tumor Immune Evasion and Metastasis by Controlling Secretion or Degradation of Multivesicular Bodies,” Developmental Cell 60, no. 15 (2025): 2114–2130. 10.1016/j.devcel.2025.03.008 40185104

[47] V. R. Penchev , Y.‐T. Chang , A. Begum , et al., “Ezrin Promotes Stem Cell Properties in Pancreatic Ductal Adenocarcinoma,” Molecular Cancer Research 17 (2019): 929–936. 10.1158/1541-7786.MCR-18-0367 PMC6445733 30655325

[48] H. Zheng , M. Wang , S. Zhang , et al., “Comprehensive Pan‐cancer Analysis Reveals NUSAP1 Is a Novel Predictive Biomarker for Prognosis and Immunotherapy Response,” International Journal of Biological Sciences 19, no. 14 (2023): 4689–4708. 10.7150/ijbs.80017 PMC10535697 37781040

[49] S. Parbin , N. Pradhan , L. Das , et al., “DNA Methylation Regulates Microtubule‐associated Tumor Suppressor 1 in human Non‐small Cell Lung Carcinoma,” Experimental Cell Research 374, no. 2 (2019): 323–332. 10.1016/j.yexcr.2018.12.004 30528566

[50] Y. Rui , H. Zhang , K. Yu , et al., “N 6 ‐Methyladenosine Regulates Cilia Elongation in Cancer Cells by Modulating HDAC6 Expression,” Advanced Science (Weinheim, Baden‐Wurttemberg, Germany) 12, no. 2 (2025): e2408488. 10.1002/advs.202408488 PMC11727115 39535388

[51] S. Han , H. Fan , G. Zhong , et al., “Nuclear KRT19 Is a Transcriptional Corepressor Promoting Histone Deacetylation and Liver Tumorigenesis,” Hepatology (Baltimore, Md) 81, no. 3 (2025): 808–822. 10.1097/HEP.0000000000000875 38557414

[52] S. K. Saha , K. Kim , G.‐M. Yang , H. Y. Choi , and S.‐G. Cho , “Cytokeratin 19 (KRT19) Has a Role in the Reprogramming of Cancer Stem Cell‐Like Cells to Less Aggressive and More Drug‐Sensitive Cells,” International Journal of Molecular Sciences 19, no. 5 (2018): 1423. 10.3390/ijms19051423 PMC5983664 29747452

[53] X. Yuan , M. Yi , B. Dong , Q. Chu , and K. Wu , “Prognostic Significance of KRT19 in Lung Squamous Cancer,” Journal of Cancer 12, no. 4 (2021): 1240–1248. 10.7150/jca.51179 PMC7797641 33442422

[54] Q. Chen , X.‐W. Zhou , A.‐J. Zhang , and K. He , “ACTN1 supports Tumor Growth by Inhibiting Hippo Signaling in Hepatocellular Carcinoma,” Journal of Experimental & Clinical Cancer Research 40, no. 1 (2021): 23. 10.1186/s13046-020-01821-6 PMC7791991 33413564

[55] Y. Sun , H. Wei , W. Yu , et al., “The Actin‐binding Protein Drebrin Disrupts NF2‐LATS Kinases Complex Assembly to Facilitate Liver Tumorigenesis,” Hepatology (Baltimore, Md) 81, no. 5 (2025): 1433–1451. 10.1097/HEP.0000000000001063 39325963

[56] S.‐E. Chow , C.‐C. Hsu , C.‐T. Yang , and Y.‐J. J. Meir , “YAP co‐Localizes with the Mitotic Spindle and Midbody to Safeguard Mitotic Division in Lung‐Cancer Cells,” The FEBS Journal 290, no. 24 (2023): 5704–5719. 10.1111/febs.16926 37549045

[57] N. Deng , W. Kang , J. Du , X. Rong , and Q. Han , “Microtubule Associated Serine/Threonine Kinase‐3 Inhibits the Malignant Phenotype of Breast Cancer by Promoting Phosphorylation‐mediated Ubiquitination Degradation of Yes‐associated Protein,” Breast Cancer Research 27, no. 1 (2025): 63. 10.1186/s13058-025-02028-3 PMC12044935 40312366

[58] J. Zhou , Y. Zeng , L. Cui , et al., “Zyxin Promotes Colon Cancer Tumorigenesis in a Mitotic Phosphorylation‐dependent Manner and through CDK8‐mediated YAP Activation,” Proceedings of the National Academy of Sciences of the United States of America 115, no. 29 (2018): e6760–e6769. 10.1073/pnas.1800621115 PMC6055133 29967145

[59] R. Chen , J. Liu , J. Hu , C. Li , Y. Liu , and W. Pan , “DLGAP5 knockdown Inactivates the Wnt/β‐Catenin Signal to Repress Endometrial Cancer Cell Malignant Activities,” Environmental Toxicology 38, no. 3 (2023): 685–693. 10.1002/tox.23720 36454672

[60] P. Zhan , B. Zhang , G.‐M. Xi , et al., “PRC1 contributes to Tumorigenesis of Lung Adenocarcinoma in Association with the Wnt/β‐catenin Signaling Pathway,” Molecular Cancer 16, no. 1 (2017): 108. 10.1186/s12943-017-0682-z PMC5483280 28646916

[61] P. Li , Y. Zhao , M. Lu , et al., “Pharmacological Inhibition of PLK1/PRC1 Triggers Mitotic Catastrophe and Sensitizes Lung Cancers to Chemotherapy,” Cell Death & Disease 16, no. 1 (2025): 374. 10.1038/s41419-025-07708-8 PMC12069692 40355412

[62] J. Ouyang , Y. Zhang , X. Hou , et al., “Long Non‐coding RNA AFAP1‐AS1 Promotes Alternative Splicing of AXIN2 by Facilitating SRSFs Phase Separation to Induce Drug Resistance in Lung Adenocarcinoma,” Molecular Cancer 24, no. 1 (2025): 294. 10.1186/s12943-025-02425-4 PMC12632050 41261425

[63] Y. Y. Zhang , H. Tabataba , X. Y. Liu , et al., “ACTN4 regulates the Stability of RIPK1 in Melanoma,” Oncogene 37, no. 29 (2018): 4033–4045. 10.1038/s41388-018-0260-x 29706658

[64] W. Zhao , C. Qi , Y. Mao , et al., “High MICAL‐L2 Promotes Cancer Progression and Drug Resistance in Renal Clear Cell Carcinoma Cells through Stabilization of ACTN4 Following Vimentin Expression,” Biochimica et Biophysica Acta (BBA)—Molecular Basis of Disease 1871, no. 3 (2025): 167628. 10.1016/j.bbadis.2024.167628 39689763

[65] Y. Zhao , T. Yu , N. Zhang , et al., “Nuclear E‐Cadherin Acetylation Promotes Colorectal Tumorigenesis via Enhancing β‐Catenin Activity,” Molecular Cancer Research 17, no. 2 (2019): 655–665. 10.1158/1541-7786.MCR-18-0637 30401720

[66] L. F. Chávez , K. Schweitzer , E. G. Alonso , et al., “GEF‐H1 Drives Breast Cancer Cells to Tumor Progression,” Biochimica et Biophysica Acta (BBA)—Molecular Basis of Disease 1871, no. 5 (2025): 167816. 10.1016/j.bbadis.2025.167816 40154811

[67] F. Hao , Y. Mou , L. Zhang , S. Wang , and Y. Yang , “LncRNA AFAP1‐AS1 Is a Prognostic Biomarker and Serves as Oncogenic Role in Retinoblastoma,” Bioscience Reports 38, no. 3 (2018): BSR20180384. 10.1042/BSR20180384 PMC6048204 29654169

[68] S. Abdul , A. Majid , J. Wang , Q. Liu , M.‐Z. Sun , and S. Liu , “Bidirectional Interaction of lncRNA AFAP1‐AS1 and CRKL Accelerates the Proliferative and Metastatic Abilities of Hepatocarcinoma Cells,” Journal of Advanced Research 24 (2020): 121–130. 10.1016/j.jare.2020.03.010 PMC7139140 32280542

[69] J. Lu , L. Zhang , H. Zhou , et al., “Silencing of Girdin Suppresses the Malignant Behavior of Colorectal Carcinoma Cells,” Oncology Reports 40, no. 2 (2018): 887–894. 10.3892/or.2018.6511 29989653

[70] X. Liu , W. Zhang , H. Wang , and W. Yang , “Identification of CKAP2 as a Potential Target for Prevention of Gastric Cancer Progression: a Multi‐Omics Study,” International Journal of Molecular Sciences 26, no. 4 (2025): 1557. 10.3390/ijms26041557 PMC11855583 40004022

[71] C. Kajiwara , K. Fumoto , H. Kimura , et al., “p63‐Dependent Dickkopf3 Expression Promotes Esophageal Cancer Cell Proliferation via CKAP4,” Cancer Research 78, no. 21 (2018): 6107–6120. 10.1158/0008-5472.CAN-18-1749 30181180

[72] A. Nagoya , R. Sada , H. Kimura , et al., “CKAP4 is a Potential Exosomal Biomarker and Therapeutic Target for Lung Cancer,” Translational Lung Cancer Research 12, no. 3 (2023): 408–426. 10.21037/tlcr-22-571 PMC10087988 37057110

[73] T. Luo , K. Ding , J. Ji , et al., “Cytoskeleton‐associated Protein 4 (CKAP4) Promotes Malignant Progression of human Gliomas through Inhibition of the Hippo Signaling Pathway,” Journal of Neuro‐oncology 154, no. 3 (2021): 275–283. 10.1007/s11060-021-03831-6 34476666

[74] H. Kimura , K. Fumoto , K. Shojima , et al., “CKAP4 is a Dickkopf1 Receptor and is Involved in Tumor Progression,” Journal of Clinical Investigation 126, no. 7 (2016): 2689–2705. 10.1172/JCI84658 PMC4922689 27322059

[75] Q.‐R. Chen , J. Liu , B.‐B. Zheng , Y.‐H. Zhu , and J.‐C. Li , “CKAP5 promotes Progression and Cisplatin Resistance in Esophageal Squamous Cell Carcinoma via Microtubule‐mediated YAP Activation,” Life Sciences 376 (2025): 123724. 10.1016/j.lfs.2025.123724 40404119

[76] L. Xia , X. Xiao , W. L. Liu , et al., “Coactosin‐Like Protein CLP/Cotl1 Suppresses Breast Cancer Growth through Activation of IL‐24/PERP and Inhibition of Non‐canonical TGFβ Signaling,” Oncogene 37, no. 3 (2018): 323–331. 10.1038/onc.2017.342 28925397

[77] A. Wang , H. Dai , Y. Gong , et al., “ANLN‐induced EZH2 Upregulation Promotes Pancreatic Cancer Progression by Mediating miR‐218‐5p/LASP1 Signaling Axis,” Journal of Experimental & Clinical Cancer Research: CR 38, no. 1 (2019): 347. 10.1186/s13046-019-1340-7 PMC6686567 31395079

[78] X. Yin , J. Wang , Z. Shen , et al., “ENAH Transcriptionally Activated by YY1 Promotes Growth and Invasion of Laryngocarcinoma Cells through PI3K/AKT Signaling,” European Journal of Pharmacology 983 (2024): 176991. 10.1016/j.ejphar.2024.176991 39265883

[79] M. Panagiotakopoulou , M. Bergert , A. Taubenberger , J. Guck , D. Poulikakos , and A. Ferrari , “A Nanoprinted Model of Interstitial Cancer Migration Reveals a Link between Cell Deformability and Proliferation,” ACS Nano 10, no. 7 (2016): 6437–6448. 10.1021/acsnano.5b07406 27268411

[80] M. Polycarpou‐Schwarz , M. Groß , P. Mestdagh , et al., “The Cancer‐associated Microprotein CASIMO1 Controls Cell Proliferation and Interacts with Squalene Epoxidase Modulating Lipid Droplet Formation,” Oncogene 37, no. 34 (2018): 4750–4768. 10.1038/s41388-018-0281-5 29765154

[81] B. Yi , Q. Fu , Z. Zheng , et al., “Pan‐cancer Analysis Reveals the Prognostic and Immunotherapeutic Value of Cytoskeleton‐associated Protein 2‐Like,” Scientific Reports 13, no. 1 (2023): 8368. 10.1038/s41598-023-35633-3 PMC10209194 37225919

[82] J. X. Xiao , W. Xu , X. Fei , et al., “Anillin Facilitates Cell Proliferation and Induces Tumor Growth of Hepatocellular Carcinoma via miR‐138/SOX4 Axis Regulation,” Translational Oncology 13, no. 10 (2020): 100815. 10.1016/j.tranon.2020.100815 PMC7341449 32645689

[83] K. Magnusson , G. Gremel , L. Rydén , et al., “ANLN Is a Prognostic Biomarker Independent of Ki‐67 and Essential for Cell Cycle Progression in Primary Breast Cancer,” BMC Cancer 16, no. 1 (2016): 904. 10.1186/s12885-016-2923-8 PMC5116155 27863473

[84] A. Bossan , R. Ottman , T. Andl , et al., “Expression of FGD4 Positively Correlates with the Aggressive Phenotype of Prostate Cancer,” BMC Cancer 18, no. 1 (2018): 1257. 10.1186/s12885-018-5096-9 PMC6296060 30558664

[85] J. Zhou , M. Liu , Y. Chen , S. Xu , Y. Guo , and L. Zhao , “Cucurbitacin B Suppresses Proliferation of Pancreatic Cancer Cells by ceRNA: Effect of miR‐146b‐5p and lncRNA‐AFAP1‐AS1,” Journal of Cellular Physiology 234, no. 4 (2019): 4655–4667. 10.1002/jcp.27264 30206930

[86] E. Piccinno , V. Scalavino , N. Labarile , et al., “miR‐195‐5p Suppresses KRT80 Expression Inducing Cell Cycle Arrest in Colon Cancer,” Cancers 17, no. 13 (2025): 2183. 10.3390/cancers17132183 PMC12248558 40647481

[87] F. Niu , Y. Liu , Z. Jing , et al., “Novel Carbazole Sulfonamide Microtubule‐destabilizing Agents Exert Potent Antitumor Activity against Esophageal Squamous Cell Carcinoma,” Cancer Letters 420 (2018): 60–71. 10.1016/j.canlet.2018.01.066 29408653

[88] S. Wang , J. Huang , C. Li , et al., “MAP9 Loss Triggers Chromosomal Instability, Initiates Colorectal Tumorigenesis, and Is Associated with Poor Survival of Patients with Colorectal Cancer,” Clinical Cancer Research 26, no. 3 (2020): 746–757. 10.1158/1078-0432.CCR-19-1611 31662330

[89] P. Liu , J. Yu , X. Tian , et al., “The Effect of Downregulation of Stathmin Gene on Biological Behaviors of U373 and U87‐MG Glioblastoma Cells,” Biological Research 51, no. 1 (2018): 16. 10.1186/s40659-018-0160-0 PMC5992777 29880026

[90] Y. Wang , F. Shi , G.‐H. Xing , et al., “Protein Regulator of Cytokinesis PRC1 Confers Chemoresistance and Predicts an Unfavorable Postoperative Survival of Hepatocellular Carcinoma Patients,” Journal of Cancer 8, no. 5 (2017): 801–808. 10.7150/jca.17640 PMC5381168 28382142

[91] A. Nurmagambetova , V. Mustyatsa , A. Saidova , and I. Vorobjev , “Morphological and Cytoskeleton Changes in Cells after EMT,” Scientific Reports 13, no. 1 (2023): 22164. 10.1038/s41598-023-48279-y PMC10719275 38092761

[92] A. M. Richardson , L. S. Havel , A. E. Koyen , et al., “Vimentin Is Required for Lung Adenocarcinoma Metastasis via Heterotypic Tumor Cell–Cancer‐Associated Fibroblast Interactions during Collective Invasion,” Clinical Cancer Research 24, no. 2 (2018): 420–432. 10.1158/1078-0432.CCR-17-1776 PMC5771825 29208669

[93] F. Wang , M. Rong , L. Zhang , et al., “Vimentin Intermediate filaments Orchestrate DNA Nonhomologous End Joining Repair and Lipolysis after DNA Damage,” Oncogene 44, no. 33 (2025): 3025–3036. 10.1038/s41388-025-03465-2 40550847

[94] C. Velez‐delValle , M. Marsch‐Moreno , F. Castro‐Muñozledo , I. J. Galván‐Mendoza , and W. Kuri‐Harcuch , “Epithelial Cell Migration Requires the Interaction between the Vimentin and Keratin Intermediate filaments,” Scientific Reports 6 (2016): 24389. 10.1038/srep24389 PMC4829867 27072292

[95] J. Fang , H. Wang , Y. Liu , F. Ding , Y. Ni , and S. Shao , “High KRT8 Expression Promotes Tumor Progression and Metastasis of Gastric Cancer,” Cancer Science 108, no. 2 (2017): 178–186. 10.1111/cas.13120 PMC5329158 27865045

[96] M. Li , X. Rao , Y. Cui , et al., “The Keratin 17/YAP/IL6 Axis Contributes to E‐cadherin Loss and Aggressiveness of Diffuse Gastric Cancer,” Oncogene 41, no. 6 (2022): 770–781. 10.1038/s41388-021-02119-3 34845376

[97] N. Guedj , J. Vaquero , A. Clapéron , et al., “Loss of Ezrin in human Intrahepatic Cholangiocarcinoma Is Associated with Ectopic Expression of E‐Cadherin,” Histopathology 69, no. 2 (2016): 211–221. 10.1111/his.12931 26791814

[98] Z. Chen , S. He , Y. Zhan , et al., “TGF‐β‐induced Transgelin Promotes Bladder Cancer Metastasis by Regulating Epithelial‐mesenchymal Transition and Invadopodia Formation,” EBioMedicine 47 (2019): 208–220. 10.1016/j.ebiom.2019.08.012 PMC6796540 31420300

[99] K. Wieczorek , M. Wiktorska , I. Sacewicz‐Hofman , et al., “Filamin A Upregulation Correlates with Snail‐induced Epithelial to Mesenchymal Transition (EMT) and Cell Adhesion but Its Inhibition Increases the Migration of Colon Adenocarcinoma HT29 Cells,” Experimental Cell Research 359, no. 1 (2017): 163–170. 10.1016/j.yexcr.2017.07.035 28778796

[100] B. C. Tröster , M. Kappelmann‐Fenzl , A. K. Bosserhoff , et al., “Emerging Role of ARHGAP29 in Melanoma Cell Phenotype Switching,” Molecular Oncology 20, no. 2 (2026): 348–368. 10.1002/1878-0261.70114 PMC12936434 40906537

[101] L.‐L. Lin , F. Yang , D.‐H. Zhang , C. Hu , S. Yang , and X.‐Q. Chen , “ARHGAP10 inhibits the Epithelial–mesenchymal Transition of Non‐small Cell Lung Cancer by Inactivating PI3K/Akt/GSK3β Signaling Pathway,” Cancer Cell International 21, no. 1 (2021): 320. 10.1186/s12935-021-02022-7 PMC8236192 34174897

[102] A. C. M. Sousa‐Squiavinato , M. R. Rocha , P. Barcellos‐de‐Souza , W. F. de Souza , and J. A. Morgado‐Diaz , “Cofilin‐1 Signaling Mediates Epithelial‐mesenchymal Transition by Promoting Actin Cytoskeleton Reorganization and Cell‐cell Adhesion Regulation in Colorectal Cancer Cells,” Biochimica et Biophysica Acta (BBA)—Molecular Cell Research 1866, no. 3 (2019): 418–429. 10.1016/j.bbamcr.2018.10.003 30296500

[103] P. Hu , B. Zong , Q. Chen , et al., “Microtubule‐associated Protein 4 Promotes Epithelial Mesenchymal Transition in Hepatocellular Cancer Cells via Regulating GSK3β/β‐catenin Pathway,” Heliyon 9, no. 3 (2023): e14309. 10.1016/j.heliyon.2023.e14309 PMC10020083 36938447

[104] M. Siatkowska , K. Robaszkiewicz , A. Rousová , et al., “Tropomyosin Isoforms Encoded by TPM2 Control the Actin‐bundling Activity of Fascin‐1,” Biological Research 58, no. 1 (2025): 60. 10.1186/s40659-025-00640-3 PMC12399007 40887664

[105] F.‐J. Zhong , B. Sun , M.‐M. Cao , C. Xu , Y.‐M. Li , and L.‐Y. Yang , “STMN2 mediates Nuclear Translocation of Smad2/3 and Enhances TGFβ Signaling by Destabilizing Microtubules to Promote Epithelial‐mesenchymal Transition in Hepatocellular Carcinoma,” Cancer Letters 506 (2021): 128–141. 10.1016/j.canlet.2021.03.001 33705863

[106] M. Shao , L. Wang , Q. Zhang , et al., “STMN2 overexpression Promotes Cell Proliferation and EMT in Pancreatic Cancer Mediated by WNT/β‐catenin Signaling,” Cancer Gene Therapy 30, no. 3 (2023): 472–480. 10.1038/s41417-022-00568-w 36460804

[107] Y. Chen , Q. Zhang , C. Ding , X. Zhang , X. Qiu , and Z. Zhang , “Stathmin1 overexpression in Hypopharyngeal Squamous Cell Carcinoma: a New Promoter in FaDu Cell Proliferation and Migration,” International Journal of Oncology 50, no. 1 (2017): 31–40. 10.3892/ijo.2016.3778 PMC5182004 27878293

[108] Y. Zhang , X. Qin , R. Guo , et al., “Notch‐1 Regulates Collective Breast Cancer Cell Migration by Controlling Intercellular Junction and Cytoskeletal Organization,” Cell Proliferation 58, no. 2 (2025): e13754. 10.1111/cpr.13754 PMC11839191 39343985

[109] M. Velázquez‐Avila , J. C. Balandrán , D. Ramírez‐Ramírez , et al., “High Cortactin Expression in B‐cell Acute Lymphoblastic Leukemia Is Associated with Increased Transendothelial Migration and Bone Marrow Relapse,” Leukemia 33, no. 6 (2019): 1337–1348. 10.1038/s41375-018-0333-4 PMC6756064 30573781

[110] S. M. Markwell , A. G. Ammer , E. T. Interval , et al., “Cortactin Phosphorylation by Casein Kinase 2 Regulates Actin‐Related Protein 2/3 Complex Activity, Invadopodia Function, and Tumor Cell Invasion,” Molecular Cancer Research 17, no. 4 (2019): 987–1001. 10.1158/1541-7786.MCR-18-0391 PMC6445698 30610108

[111] Z. Cheng , W. Wei , Z. Wu , et al., “ARPC2 promotes Breast Cancer Proliferation and Metastasis,” Oncology Reports 41, no. 6 (2019): 3189–3200. 10.3892/or.2019.7113 PMC6488984 31002363

[112] Y. Sun , Y. Qiao , Y. Niu , et al., “ARP2/3 complex Affects Myofibroblast Differentiation and Migration in Pancreatic Ductal Adenocarcinoma,” International Journal of Cancer 156, no. 6 (2025): 1272–1281. 10.1002/ijc.35246 PMC11737003 39472297

[113] K. Taniuchi , M. Furihata , S. Naganuma , et al., “WAVE2 is Associated with Poor Prognosis in Pancreatic Cancers and Promotes Cell Motility and Invasiveness via Binding to ACTN4,” Cancer Medicine 7, no. 11 (2018): 5733–5751. 10.1002/cam4.1837 PMC6246955 30353690

[114] N. Z. Jäntti , P. Moreno‐Layseca , M. R. Chastney , et al., “EPLINα Controls Integrin Recycling from Rab21 Endosomes to Drive Breast Cancer Cell Migration,” Developmental Cell 60, no. 22 (2025): 3018–3033. 10.1016/j.devcel.2025.06.025 40669465

[115] R. Sargent , D. H. Liu , R. Yadav , et al., “Integrated Structural Model of the Palladin–actin Complex Using XL ‐ MS , Docking, NMR , and SAXS,” Protein Science 34, no. 5 (2025): e70122. 10.1002/pro.70122 PMC12006749 40248864

[116] M. Seong , P. Bak‐Gordon , Z. Liu , P. D. Canoll , and J. L. Manley , “Splicing Dysregulation in Glioblastoma Alters the Function of Cell Migration‐Related Genes,” Glia 73, no. 2 (2025): 251–270. 10.1002/glia.24630 PMC11663109 39448549

[117] H. Wu , R. Hasan , H. Zhang , et al., “Phosphorylation Regulates CAP1 (Cyclase‐Associated Protein 1) Functions in the Motility and Invasion of Pancreatic Cancer Cells,” Scientific Reports 9, no. 1 (2019): 4925. 10.1038/s41598-019-41346-3 PMC6426867 30894654

[118] M. Pan , T. W. Chew , D. C. P. Wong , et al., “BNIP‐2 Retards Breast Cancer Cell Migration by Coupling Microtubule‐mediated GEF‐H1 and RhoA Activation,” Science Advances 6, no. 31 (2020): eaaz1534. 10.1126/sciadv.aaz1534 PMC7399486 32789168

[119] C. Gullo , E. Laverdeur , M. Dancre , et al., “Focal Adhesion Assembly and Cell Migration Require Myoferlin in PDAC Cell Lines,” Scientific Reports 15, no. 1 (2025): 42797. 10.1038/s41598-025-27134-2 PMC12663238 41315407

[120] Y. Wang , N. A. Jerome , A. J. Kelleher , et al., “TRIM29 promotes Bladder Cancer Invasion by Regulating the Intermediate Filament Network and Focal Adhesion,” Oncogene 44, no. 42 (2025): 4047–4057. 10.1038/s41388-025-03557-z PMC12518127 40908312

[121] J. M. Mondaca , J. M. F. Muñoz , G. A. Barraza , et al., “Therapeutic Potential of GNRHR Analogs and SRC/FAK Inhibitors to Counteract Tumor Growth and Metastasis in Breast Cancer,” Biochimica et Biophysica Acta (BBA)—Molecular Basis of Disease 1871, no. 5 (2025): 167826. 10.1016/j.bbadis.2025.167826 40189112

[122] X. Lei , L. Deng , D. Liu , et al., “ARHGEF7 promotes Metastasis of Colorectal Adenocarcinoma by Regulating the Motility of Cancer Cells,” International Journal of Oncology 53, no. 5 (2018): 1980–1996. 10.3892/ijo.2018.4535 PMC6192735 30132516

[123] A. G. Casanova , G. S. Roth , S. Hausmann , et al., “Cytoskeleton Remodeling Induced by SMYD2 Methyltransferase Drives Breast Cancer Metastasis,” Cell Discovery 10, no. 1 (2024): 12. 10.1038/s41421-023-00644-x PMC10830559 38296970

[124] X. Huang , Z. Li , Y. Huang , et al., “Vimentin Intermediate filaments Coordinate Actin Stress Fibers and Podosomes to Determine the Extracellular Matrix Degradation by Macrophages,” Developmental Cell 60, no. 12 (2025): 1669–1685. 10.1016/j.devcel.2025.01.016 39952241

[125] D. Xu , X. Zhou , S. Min , et al., “Leukocyte‐specific Protein 1 Is Associated with the Stage and Tumor Immune Infiltration of Cervical Cancer,” Scientific Reports 15, no. 1 (2025): 7566. 10.1038/s41598-025-91066-0 PMC11880245 40038352

[126] A. Lahusen , N. Minhöfer , K.‐A. Lohse , et al., “Pancreatic Cancer Cell‐intrinsic Transglutaminase‐2 Promotes T Cell Suppression through Microtubule‐dependent Secretion of Immunosuppressive Cytokines,” Journal for Immunotherapy of Cancer 13, no. 1 (2025): e010579. 10.1136/jitc-2024-010579 PMC11748943 39824529

[127] G. Li , Y. Zhong , Q. Shen , et al., “NEK2 serves as a Prognostic Biomarker for Hepatocellular Carcinoma,” International Journal of Oncology 50, no. 2 (2017): 405–413. 10.3892/ijo.2017.3837 PMC5238800 28101574

[128] S. M. Goicoechea , A. Zinn , S. S. Awadia , K. Snyder , and R. Garcia‐Mata , “A RhoG‐mediated Signaling Pathway That Modulates Invadopodia Dynamics in Breast Cancer Cells,” Journal of Cell Science 130, no. 6 (2017): 1064–1077. 10.1242/jcs.195552 PMC5358339 28202690

[129] F. S. Guerra , R. G. D. Oliveira , C. A. M. Fraga , C. D. S. Mermelstein , and P. D. Fernandes , “ROCK Inhibition with Fasudil Induces Beta‐catenin Nuclear Translocation and Inhibits Cell Migration of MDA‐MB 231 human Breast Cancer Cells,” Scientific Reports 7, no. 1 (2017): 13723. 10.1038/s41598-017-14216-z PMC5651822 29057980

[130] Z. Wei , J. Li , L. Zhong , et al., “DDR1 Drives Malignant Progression of Gastric Cancer by Suppressing HIF‐1α Ubiquitination and Degradation,” Advanced Science (Weinheim, Baden‐Wurttemberg, Germany) 11, no. 35 (2024): e2308395. 10.1002/advs.202308395 PMC11425230 39024501

[131] C. M. Fife , S. M. Sagnella , W. S. Teo , et al., “Stathmin Mediates Neuroblastoma Metastasis in a Tubulin‐independent Manner via RhoA/ROCK Signaling and Enhanced Transendothelial Migration,” Oncogene 36, no. 4 (2017): 501–511. 10.1038/onc.2016.220 27321182

[132] X. Lin , Z. Zhao , S.‐P. Sun , and W. Liu , “Scinderin Promotes Glioma Cell Migration and Invasion via Remodeling Actin Cytoskeleton,” World Journal of Clinical Oncology 15, no. 1 (2024): 32–44. 10.5306/wjco.v15.i1.32 PMC10823943 38292665

[133] J.‐H. Choi , J. Jeong , J. Kim , et al., “Genetic Disruption of ATAT1 Causes RhoA Downregulation through Abnormal Truncation of C/EBPβ,” BMB Reports 57, no. 6 (2024): 293–298. 10.5483/BMBRep.2023-0230 PMC11214891 38835115

[134] Q. Chen , P. Wu , J. Cai , et al., “A Moonlighting Function of Tumoral Interleukin‐1β Precursor Promotes Metastasis via RACK1‐mediated Actin Remodeling,” Nature Communications 16, no. 1 (2025): 10148. 10.1038/s41467-025-65220-1 PMC12630722 41257838

[135] L. Chen , J. Cai , Y. Huang , et al., “Identification of Cofilin‐1 as a Novel Mediator for the Metastatic Potentials and Chemoresistance of the Prostate Cancer Cells,” European Journal of Pharmacology 880 (2020): 173100. 10.1016/j.ejphar.2020.173100 32320704

[136] L. Zeng , X. Lyu , J. Yuan , et al., “STMN1 Promotes Tumor Metastasis in Non‐small Cell Lung Cancer through Microtubule‐dependent and Nonmicrotubule‐dependent Pathways,” International Journal of Biological Sciences 20, no. 4 (2024): 1509–1527. 10.7150/ijbs.84738 PMC10878155 38385074

[137] L. Fu , W. Liao , Y. Tan , et al., “TWF2 Drives Tumor Progression and Sunitinib Resistance in Renal Cell Carcinoma through Hippo Signaling Suppression,” Advanced Science (Weinheim, Baden‐Wurttemberg, Germany) 12, no. 44 (2025): e06367. 10.1002/advs.202506367 PMC12667553 40948085

[138] Y. Ye , F. Shen , M. Yan , et al., “Intermediate Filament IFFO1 Negatively Regulates the Migration of Lung Cancer Cells by Inhibiting the IQGAP3‐Cdc42 Interaction,” Cell Death & Disease 16, no. 1 (2025): 509. 10.1038/s41419-025-07846-z PMC12241364 40634295

[139] L. Chen , C. He , Z. Ou , and C. Zhao , “TNF‐α Drives Bladder Cancer Metastasis via METTL3‐mediated m6A Modification to Promote CLASP2/IQGAP1‐dependent Cytoskeleton Remodeling,” Biochimica et Biophysica Acta (BBA)—Molecular Basis of Disease 1871, no. 5 (2025): 167811. 10.1016/j.bbadis.2025.167811 40118293

[140] Y.‐Y. Jiang , L. Shang , Z.‐Z. Shi , et al., “Microtubule‐associated Protein 4 Is an Important Regulator of Cell Invasion/Migration and a Potential Therapeutic Target in Esophageal Squamous Cell Carcinoma,” Oncogene 35, no. 37 (2016): 4846–4856. 10.1038/onc.2016.17 26876215

[141] I. Shapiro , H. Debaix , C. Kräuchi , et al., “(Non)canonical Wnt Signaling, Cytoarchitecture and Stemness: New Insights from Primary Nonmetastatic, Primary Metastatic, Regional and Distant Metastatic Models of Adrenocortical Carcinoma,” Experimental & Molecular Medicine 57, no. 11 (2025): 2657–2670. 10.1038/s12276-025-01507-z PMC12686465 41310103

[142] Y. Cheng , K. Qin , N. Huang , et al., “Cytokeratin 18 Regulates the Transcription and Alternative Splicing of Apoptotic‑Related Genes and Pathways in HeLa Cells,” Oncology Reports 42, no. 1 (2019): 301–312. 10.3892/or.2019.7166 PMC6549092 31115582

[143] D. Guo , Q. Xu , S. Pabla , et al., “Cytokeratin‐8 in Anaplastic Thyroid Carcinoma: More than a Simple Structural Cytoskeletal Protein,” International Journal of Molecular Sciences 19, no. 2 (2018): 577. 10.3390/ijms19020577 PMC5855799 29443941

[144] S. K. Trisdale , N. M. Schwab , X. Hou , J. S. Davis , and D. H. Townson , “Molecular Manipulation of Keratin 8/18 Intermediate filaments: Modulators of FAS‐mediated Death Signaling in human Ovarian Granulosa Tumor Cells,” Journal of Ovarian Research 9 (2016): 8. 10.1186/s13048-016-0217-z PMC4765146 26911253

[145] L. Fang , M. Zhang , L. Chen , et al., “Downregulation of Nucleolar and Spindle‐associated Protein 1 Expression Suppresses Cell Migration, Proliferation and Invasion in Renal Cell Carcinoma,” Oncology Reports 36, no. 3 (2016): 1506–1516. 10.3892/or.2016.4955 27461786

[146] J. Zhang , J.‐Z. Huang , Y.‐Q. Zhang , et al., “Microtubule Associated Protein 9 Inhibits Liver Tumorigenesis by Suppressing ERCC3,” EBioMedicine 53 (2020): 102701. 10.1016/j.ebiom.2020.102701 PMC7063135 32151798

[147] E. Cetti , T. Di Marco , G. Mauro , et al., “Mitosis Perturbation by MASTL Depletion Impairs the Viability of Thyroid Tumor Cells,” Cancer Letters 442 (2019): 362–372. 10.1016/j.canlet.2018.11.010 30445205

[148] P. Bao , T. Yokobori , B. Altan , et al., “High STMN1 Expression Is Associated with Cancer Progression and Chemo‐Resistance in Lung Squamous Cell Carcinoma,” Annals of Surgical Oncology 24, no. 13 (2017): 4017–4024. 10.1245/s10434-017-6083-0 28933054

[149] J. Wang , Y. Yao , Y. Ming , et al., “Downregulation of Stathmin 1 in human Gallbladder Carcinoma Inhibits Tumor Growth in Vitro and in Vivo,” Scientific Reports 6 (2016): 28833. 10.1038/srep28833 PMC4923895 27349455

[150] Z. Zhang , M. Li , Y. Hou , et al., “SETD7 promotes LC3B Methylation and Degradation in Ovarian Cancer,” Journal of Biological Chemistry 301, no. 2 (2025): 108134. 10.1016/j.jbc.2024.108134 PMC11791264 39725038

[151] X. Liu , Z. Hao , H. He , et al., “Accumulation of Microtubule‐associated Protein Tau Promotes Hepatocellular Carcinogenesis through Inhibiting Autophagosome‐lysosome Fusion,” Molecular and Cellular Biochemistry 480, no. 6 (2025): 3621–3635. 10.1007/s11010-024-05193-9 39718681

[152] Y. Dong , Q. Jin , M. Sun , et al., “CLDN6 inhibits Breast Cancer Metastasis through WIP‐dependent Actin Cytoskeleton‐mediated Autophagy,” Journal of Experimental & Clinical Cancer Research 42, no. 1 (2023): 68. 10.1186/s13046-023-02644-x PMC10026481 36935496

[153] Y. Wang , R. Zuo , G. Huo , et al., “TWF1 induces Autophagy and Accelerates Malignant Phenotype in Lung Adenocarcinoma via Inhibiting the cAMP Signaling Pathway,” The FASEB Journal 37, no. 7 (2023): e23051. 10.1096/fj.202300248R 37358822

[154] Y. Ding , C. Lv , Y. Zhou , et al., “Vimentin Loss Promotes Cancer Proliferation through Up‐regulating Rictor/AKT/β‐catenin Signaling Pathway,” Experimental Cell Research 405, no. 1 (2021): 112666. 10.1016/j.yexcr.2021.112666 34052237

[155] L. Zhang , X. Liu , L. Song , H. Zhai , and C. Chang , “MAP7 promotes Migration and Invasion and Progression of human Cervical Cancer through Modulating the Autophagy,” Cancer Cell International 20 (2020): 17. 10.1186/s12935-020-1095-4 PMC6958635 31956295

[156] Y. Zhang , Z. Li , H. Lu , et al., “Pan‐cancer Analysis Uncovered the Prognostic and Therapeutic Value of Disulfidptosis,” NPJ Precision Oncology 9, no. 1 (2025): 50. 10.1038/s41698-025-00834-8 PMC11850913 39994355

[157] Y. Tan , L. Yang , Q. Wang , et al., “CD2AP is a Disulfidptosis Modulator and Prognostic Biomarker in Hepatocellular Carcinoma,” Clinical and Experimental Medicine 26, no. 1 (2025): 5. 10.1007/s10238-025-01917-3 PMC12628418 41249753

[158] J. Jang , H. J. Park , W. Seong , J. Kim , and C. Kim , “Vimentin‐mediated Buffering of Internal Integrin β1 Pool Increases Survival of Cells from Anoikis,” BMC Biology 22, no. 1 (2024): 139. 10.1186/s12915-024-01942-w PMC11197373 38915055

[159] X. Jiang , J. Hu , Z. Wu , et al., “Protein Phosphatase 2A Mediates YAP Activation in Endothelial Cells Upon VEGF Stimulation and Matrix Stiffness,” Frontiers in Cell and Developmental Biology 9 (2021): 675562. 10.3389/fcell.2021.675562 PMC8158299 34055807

[160] K. M.‐Y. Kiang , P. Zhang , N. Li , Z. Zhu , L. Jin , and G. K.‐K. Leung , “Loss of Cytoskeleton Protein ADD3 Promotes Tumor Growth and Angiogenesis in Glioblastoma Multiforme,” Cancer Letters 474 (2020): 118–126. 10.1016/j.canlet.2020.01.007 31958485

[161] J. R. van Beijnum , E. J. M. Huijbers , K. van Loon , et al., “Extracellular Vimentin Mimics VEGF and Is a Target for Anti‐angiogenic Immunotherapy,” Nature Communications 13, no. 1 (2022): 2842. 10.1038/s41467-022-30063-7 PMC9126915 35606362

[162] G. He , W. Li , W. Zhao , et al., “Formin‐like 2 Promotes Angiogenesis and Metastasis of Colorectal Cancer by Regulating the EGFL6 / CKAP4 / ERK Axis,” Cancer Science 114, no. 5 (2023): 2014–2028. 10.1111/cas.15739 PMC10154862 36715549

[163] H. Singh , A. Thirupathi , B. Das , et al., “2,3‐Difunctionalized Benzo[ b ]Thiophene Scaffolds Possessing Potent Antiangiogenic Properties,” Journal of Medicinal Chemistry 65, no. 1 (2022): 120–134. 10.1021/acs.jmedchem.1c00892 34914389

[164] N. Molinie , S. N. Rubtsova , A. Fokin , et al., “Cortical Branched Actin Determines Cell Cycle Progression,” Cell Research 29, no. 6 (2019): 432–445. 10.1038/s41422-019-0160-9 PMC6796858 30971746

[165] P. A. Janmey , D. A. Fletcher , and C. A. Reinhart‐King , “Stiffness Sensing by Cells,” Physiological Reviews 100, no. 2 (2020): 695–724. 10.1152/physrev.00013.2019 PMC7276923 31751165

[166] V. Gkretsi , A. Stylianou , and T. Stylianopoulos , “Vasodilator‐Stimulated Phosphoprotein (VASP) Depletion from Breast Cancer MDA‐MB‐231 Cells Inhibits Tumor Spheroid Invasion through Downregulation of Migfilin, β‐catenin and Urokinase‐plasminogen Activator (uPA),” Experimental Cell Research 352, no. 2 (2017): 281–292. 10.1016/j.yexcr.2017.02.019 PMC5349498 28209486

[167] S. Azadi , M. Tafazzoli‐Shadpour , M. Soleimani , and M. E. Warkiani , “Modulating Cancer Cell Mechanics and Actin Cytoskeleton Structure by Chemical and Mechanical Stimulations,” Journal of Biomedical Materials Research Part A 107, no. 8 (2019): 1569–1581. 10.1002/jbm.a.36670 30884131

[168] T. Hohmann , U. Grabiec , C. Vogel , et al., “The Impact of Non‐Lethal Single‐Dose Radiation on Tumor Invasion and Cytoskeletal Properties,” International Journal of Molecular Sciences 18, no. 9 (2017): 2001. 10.3390/ijms18092001 PMC5618650 28926987

[169] J. Wang , B. Zhang , X. Chen , et al., “Cell Mechanics Regulate the Migration and Invasion of Hepatocellular Carcinoma Cells via JNK Signaling,” Acta Biomaterialia 176 (2024): 321–333. 10.1016/j.actbio.2024.01.024 38272199

[170] X. Chen , Z. Xu , K. Tang , et al., “The Mechanics of Tumor Cells Dictate Malignancy via Cytoskeleton‐Mediated APC/Wnt/β‐Catenin Signaling,” Research (Washington, DC) 6 (2023): 0224. 10.34133/research.0224 PMC10513157 37746658

[171] D. A. Rudzka , G. Spennati , D. J. McGarry , et al., “Migration through Physical Constraints Is Enabled by MAPK‐induced Cell Softening via Actin Cytoskeleton Re‐organization,” Journal of Cell Science 132, no. 11 (2019): 224071. 10.1242/jcs.224071 PMC6589089 31152052

[172] D. S. Kang , A. Moriarty , Y. J. Wang , et al., “Ectopic Expression of a Truncated Isoform of Hair Keratin 81 in Breast Cancer Alters Biophysical Characteristics to Promote Metastatic Propensity,” Advanced Science (Weinheim, Baden‐Wurttemberg, Germany) 11, no. 5 (2024): e2300509. 10.1002/advs.202300509 PMC10837353 37949677

[173] M. Aragona , T. Panciera , A. Manfrin , et al., “A Mechanical Checkpoint Controls Multicellular Growth through YAP/TAZ Regulation by Actin‐processing Factors,” Cell 154, no. 5 (2013): 1047–1059. 10.1016/j.cell.2013.07.042 23954413

[174] A. Elosegui‐Artola , R. Oria , Y. Chen , et al., “Mechanical Regulation of a Molecular Clutch Defines Force Transmission and Transduction in Response to Matrix Rigidity,” Nature Cell Biology 18, no. 5 (2016): 540–548. 10.1038/ncb3336 27065098

[175] A. Elosegui‐Artola , I. Andreu , A. E. M. Beedle , et al., “Force Triggers YAP Nuclear Entry by Regulating Transport across Nuclear Pores,” Cell 171, no. 6 (2017): 1397–1410. 10.1016/j.cell.2017.10.008 29107331

[176] F. Bianchi , M. Sommariva , L. B. Cornaghi , et al., “Mechanical Cues, E‐Cadherin Expression and Cell “Sociality” Are Crucial Crossroads in Determining Pancreatic Ductal Adenocarcinoma Cells Behavior,” Cells 11, no. 8 (2022): 1318. 10.3390/cells11081318 PMC9028873 35455997

[177] J. Duan , H. Li , J. Zhang , et al., “PIEZO1 Affects Cell Growth and Migration via Microfilament‐Mediated YAP Trans‐Latitudinal Regulation,” Analytical Chemistry 97, no. 1 (2025): 147–156. 10.1021/acs.analchem.4c03420 39729436

[178] J. Zhou , Y. Deng , J. Mei , et al., “Polycystin‐1 Mutant Alters Mechanotransduction in Response to Collagen and Extracellular Matrix Stiffness via Daam1‐Dependent Microfilament Remodeling,” Advanced Science (Weinheim, Baden‐Wurttemberg, Germany) 12, no. 41 (2025): e09846. 10.1002/advs.202509846 PMC12591123 40789101

[179] T. Triulzi , M. Giussani , E. Maffioli , et al., “Proteomic Landscape of Decellularized Breast Carcinomas Identifies C‐type Lectin Domain family 3 Member A as a Driver of Cancer Aggressiveness,” NPJ Breast Cancer 11 (2025): 51. 10.1038/s41523-025-00769-0 PMC12144095 40480997

[180] X. Fan , H. Chen , Y. Li , et al., “Actin‐Targeted Magnetic Nanomotors Mechanically Modulate the Tumor Mechanical Microenvironment for Cancer Treatment,” ACS Nano 19, no. 6 (2025): 6454–6467. 10.1021/acsnano.4c17229 39915111

[181] G. Zhang , T. Yu , X. Chai , et al., “Gradient Rotating Magnetic Fields Impairing F‐Actin‐Related Gene CCDC150 to Inhibit Triple‐Negative Breast Cancer Metastasis by Inactivating TGF‐β1/SMAD3 Signaling Pathway,” Research (Washington, DC) 7 (2024): 0320. 10.34133/research.0320 PMC10900498 38420580

[182] K. Mandal , K. Pogoda , S. Nandi , et al., “Role of a Kinesin Motor in Cancer Cell Mechanics,” Nano Letters 19, no. 11 (2019): 7691–7702. 10.1021/acs.nanolett.9b02592 PMC7737127 31565944

[183] A. V. Nguyen , B. Trompetto , X. H. M. Tan , et al., “Differential Contributions of Actin and Myosin to the Physical Phenotypes and Invasion of Pancreatic Cancer Cells,” Cellular and Molecular Bioengineering 13, no. 1 (2020): 27–44. 10.1007/s12195-019-00603-1 PMC6981337 32030106

[184] M. B. Alvarez‐Elizondo , Y. Merkher , G. Shleifer , C. Gashri , and D. Weihs , “Actin as a Target to Reduce Cell Invasiveness in Initial Stages of Metastasis,” Annals of Biomedical Engineering 49, no. 5 (2021): 1342–1352. 10.1007/s10439-020-02679-7 33145677

[185] M. Azatov , S. M. Goicoechea , C. A. Otey , and A. Upadhyaya , “The Actin Crosslinking Protein Palladin Modulates Force Generation and Mechanosensitivity of Tumor Associated Fibroblasts,” Scientific Reports 6 (2016): 28805. 10.1038/srep28805 PMC4926206 27353427

[186] X. Zhang , G. Li , Y. Guo , et al., “Regulation of Ezrin Tension by S‐nitrosylation Mediates Non‐small Cell Lung Cancer Invasion and Metastasis,” Theranostics 9, no. 9 (2019): 2555–2571. 10.7150/thno.32479 PMC6525990 31131053

[187] A. Yamagishi , M. Susaki , Y. Takano , et al., “The Structural Function of Nestin in Cell Body Softening Is Correlated with Cancer Cell Metastasis,” International Journal of Biological Sciences 15, no. 7 (2019): 1546–1556. 10.7150/ijbs.33423 PMC6643143 31337983

[188] A. Yamagishi , R. Tokuoka , K. Imai , et al., “Nestin Forms a Flexible Cytoskeleton by Means of a Huge Tail Domain That Is Reversibly Stretched and Contracted by Weak Forces,” Cells 14, no. 2 (2025): 138. 10.3390/cells14020138 PMC11763517 39851565

[189] J. Sun , Q. Luo , L. Liu , and G. Song , “Low‐level Shear Stress Promotes Migration of Liver Cancer Stem Cells via the FAK‐ERK1/2 Signalling Pathway,” Cancer Letters 427 (2018): 1–8. 10.1016/j.canlet.2018.04.015 29678550

[190] M. Karasová , M. Jobst , D. Framke , et al., “Mechanical Cues Rewire Lipid Metabolism and Support Chemoresistance in Epithelial Ovarian Cancer Cell Lines OVCAR3 and SKOV3,” Cell Communication and Signaling: CCS 23, no. 1 (2025): 193. 10.1186/s12964-025-02144-9 PMC12016438 40264231

[191] X. Sun , Y. Zhou , S. Sun , et al., “Cancer Cells Sense Solid Stress to Enhance Metastasis by CKAP4 Phase Separation‐mediated Microtubule Branching,” Cell Discovery 10, no. 1 (2024): 114. 10.1038/s41421-024-00737-1 PMC11554681 39528501

[192] E. J. van Bodegraven , E. Infante , F. Peglion , et al., “Intermediate filaments Promote Glioblastoma Cell Invasion by Controlling Nuclear Deformations and Mechanosensitive Expression of MMP14,” Cell Reports 44, no. 11 (2025): 116553. 10.1016/j.celrep.2025.116553 41241939

[193] D. Wang , Y. Wang , X. Di , et al., “Cortical Tension Drug Screen Links Mitotic Spindle Integrity to Rho Pathway,” Current Biology 33, no. 20 (2023): 4458–4469. 10.1016/j.cub.2023.09.022 37875071

[194] M. Gharooni , A. Alikhani , H. Moghtaderi , et al., “Bioelectronics of the Cellular Cytoskeleton: Monitoring Cytoskeletal Conductance Variation for Sensing Drug Resistance,” ACS Sensors 4 (2019): 353–362. 10.1021/acssensors.8b01142 30572702

[195] A. J. Kuipers , J. Middelbeek , K. Vrenken , et al., “TRPM7 controls Mesenchymal Features of Breast Cancer Cells by Tensional Regulation of SOX4,” Biochimica et Biophysica Acta (BBA)—Molecular Basis of Disease 1864, no. 7 (2018): 2409–2419. 10.1016/j.bbadis.2018.04.017 29684587

[196] A. Saju , P.‐P. Chen , T.‐H. Weng , et al., “HURP Binding to the Vinca Domain of β‐tubulin Accounts for Cancer Drug Resistance,” Nature Communications 15, no. 1 (2024): 8844. 10.1038/s41467-024-53139-y PMC11471760 39397030

[197] S. Zhang , X. Zhang , W. Huang , et al., “NUSAP1 is Upregulated by Estrogen to Promote Lung Adenocarcinoma Growth and Serves as a Therapeutic Target,” International Journal of Biological Sciences 20, no. 13 (2024): 5375–5395. 10.7150/ijbs.100188 PMC11489181 39430250

[198] J. Yang , Y. Yu , W. Liu , Z. Li , Z. Wei , and R. Jiang , “Microtubule‐associated Protein Tau Is Associated with the Resistance to Docetaxel in Prostate Cancer Cell Lines,” Research and Reports in Urology 9 (2017): 71–77. 10.2147/RRU.S118966 PMC5428793 28507979

[199] H. Yang , J. Qin , Y. Pei , et al., “Discovery of the Cereblon‐recruiting Tubulin PROTACs Effective in Overcoming Taxol Resistance in Vitro and in Vivo,” European Journal of Medicinal Chemistry 265 (2024): 116067. 10.1016/j.ejmech.2023.116067 38171146

[200] W. Wang , H. Zhang , X. Wang , et al., “Novel Mutations Involving βI‐, βIIA‐, or βIVB‐tubulin Isotypes with Functional Resemblance to βIII‐tubulin in Breast Cancer,” Protoplasma 254, no. 3 (2017): 1163–1173. 10.1007/s00709-016-1060-1 27943021

[201] M. Schmitt , T. Sinnberg , N. C. Nalpas , A. Maass , B. Schittek , and B. Macek , “Quantitative Proteomics Links the Intermediate Filament Nestin to Resistance to Targeted BRAF Inhibition in Melanoma Cells,” Molecular & Cellular Proteomics: MCP 18, no. 6 (2019): 1096–1109. 10.1074/mcp.RA119.001302 PMC6553926 30890564

[202] S. Suzuki , T. Yokobori , B. Altan , et al., “High Stathmin 1 Expression Is Associated with Poor Prognosis and Chemoradiation Resistance in Esophageal Squamous Cell Carcinoma,” International Journal of Oncology 50, no. 4 (2017): 1184–1190. 10.3892/ijo.2017.3899 28350065

[203] J. W. Smyth , S. Guo , L. Chaunsali , et al., “Cytoplasmic connexin43‐microtubule Interactions Promote Glioblastoma Stem‐Like Cell Maintenance and Tumorigenicity,” Cell Death & Disease 16, no. 1 (2025): 388. 10.1038/s41419-025-07514-2 PMC12084297 40379630

[204] J. Yang , N. Li , X. Zhao , et al., “WP1066, a Small Molecule Inhibitor of STAT3, Chemosensitizes Paclitaxel‐resistant Ovarian Cancer Cells to Paclitaxel by Simultaneously Inhibiting the Activity of STAT3 and the Interaction of STAT3 with Stathmin,” Biochemical Pharmacology 221 (2024): 116040. 10.1016/j.bcp.2024.116040 38311257

[205] Y. Ren , X. Wang , Z. Lou , et al., “Induction of Cell Cycle Arrest by Increasing GTP‐RhoA Levels via Taxol‐induced Microtubule Polymerization in Renal Cell Carcinoma,” Molecular Medicine Reports 15, no. 6 (2017): 4273–4279. 10.3892/mmr.2017.6543 PMC5436224 28487984

[206] F. Shen , D. Long , T. Yu , et al., “Vinblastine Differs from Taxol as It Inhibits the Malignant Phenotypes of NSCLC Cells by Increasing the Phosphorylation of Op18/Stathmin,” Oncology Reports 37, no. 4 (2017): 2481–2489. 10.3892/or.2017.5469 28259950

[207] S. Song , P. Ko , S. Keum , et al., “Microtubule Acetylation and PERK Activation Facilitate Eribulin‐induced Mitochondrial Calcium Accumulation and Cell Death,” Cellular and Molecular Life Sciences 82, no. 1 (2024): 32. 10.1007/s00018-024-05565-w PMC11688268 39741209

[208] X.‐Y. He , C.‐Y. Liu , X.‐Y. Ding , et al., “KY216‐tubulin Complex Captures VASH2 to Inhibit NSCLC Metastasis,” Nature Communications 17, no. 1 (2025): 191. 10.1038/s41467-025-66817-2 PMC12780157 41345086

[209] L. Rickardson , E. Kutvonen , S. Orasniemi , et al., “Evaluation of the Antitumor Activity of NOV202, a Novel Microtubule Targeting and Vascular Disrupting Agent,” Drug Design, Development and Therapy 11 (2017): 1335–1351. 10.2147/DDDT.S133189 PMC5417661 28496304

[210] R. R. Hire , S. Srivastava , M. B. Davis , A. Kumar Konreddy , and D. Panda , “Antiproliferative Activity of Crocin Involves Targeting of Microtubules in Breast Cancer Cells,” Scientific Reports 7 (2017): 44984. 10.1038/srep44984 PMC5364484 28337976

[211] J. J. Vicente , K. Khan , G. Tillinghast , J. L. McFaline‐Figueroa , Y. Sancak , and N. Stella , “The Microtubule Targeting Agent ST‐401 Triggers Cell Death in Interphase and Prevents the Formation of Polyploid Giant Cancer Cells,” Journal of Translational Medicine 22 (2024): 441. 10.1186/s12967-024-05234-3 PMC11084142 38730481

[212] L. Lindström , J. Zhou , B. O. Villoutreix , et al., “Targeting TUBG1 in RB1 ‐Negative Tumors,” The FASEB Journal 39, no. 5 (2025): e70431. 10.1096/fj.202403180RR PMC11869804 40019206

[213] B. Jiang , Y. Zheng , T. Xue , et al., “Identification of Selenium‐containing Benzamides as Potent Microtubule‐targeting Antitumor Agents,” Bioorganic Chemistry 159 (2025): 108355. 10.1016/j.bioorg.2025.108355 40090150

[214] X. Wang , Y. Wang , X. Wang , Y. Wang , and Q. Liu , “Discovery of the Erianin Derivatives as EGFR/Tubulin Dual‐target Inhibitors That Suppress the Proliferation and Invasion of Non‐small Cell Lung Cancer through Autophagy‐dependent Ferroptosis,” Bioorganic chemistry 166 (2025): 109110. 10.1016/j.bioorg.2025.109110 41110239

[215] H. Amawi , N. A. Hussein , C. R. Ashby , et al., “Bax/Tubulin/Epithelial‐Mesenchymal Pathways Determine the Efficacy of Silybin Analog HM015k in Colorectal Cancer Cell Growth and Metastasis,” Frontiers in Pharmacology 9 (2018): 520. 10.3389/fphar.2018.00520 PMC5974752 29875662

[216] J.‐W. Ji , X.‐J. Liu , J. Wu , et al., “Discovery of Novel 1,2,3‐triazole Arylamide Derivatives Bearing Dithiocarbamate Moiety as Dual Inhibitors of Tubulin and LSD1 with Potent Anticancer Activity,” European Journal of Medicinal Chemistry 296 (2025): 117879. 10.1016/j.ejmech.2025.117879 40582186

[217] Y. Guo , Q. Yu , C. Guo , et al., “Structure‐guided Design of Potent Tetrazolo[1,5‐a]Pyrimidine‐based Tubulin Inhibitors with in Vivo Antitumor Activity,” European Journal of Medicinal Chemistry 302, no. Pt 1 (2026): 118288. 10.1016/j.ejmech.2025.118288 41172644

[218] W.‐B. Liu , W.‐G. Yang , J. Wu , et al., “Discovery of Novel Thienopyridine Indole Derivatives as Inhibitors of Tubulin Polymerization Targeting the Colchicine‐binding Site with Potent Anticancer Activities,” European Journal of Medicinal Chemistry 286 (2025): 117314. 10.1016/j.ejmech.2025.117314 39874633

[219] P. Shan , K.‐L. Liu , X. Jiang , G. Zhou , K. Zhu , and H. Zhang , “Discovery of a Novel Potent Tubulin Inhibitor through Virtual Screening and Target Validation for Cancer Chemotherapy,” Cell Death Discovery 11, no. 1 (2025): 392. 10.1038/s41420-025-02679-3 PMC12365163 40830098

[220] R. Romagnoli , F. Prencipe , P. Oliva , et al., “3‐Aryl/Heteroaryl‐5‐amino‐1‐(3′,4′,5′‐trimethoxybenzoyl)‐1,2,4‐triazoles as Antimicrotubule Agents. Design, Synthesis, Antiproliferative Activity and Inhibition of Tubulin Polymerization,” Bioorganic Chemistry 80 (2018): 361–374. 10.1016/j.bioorg.2018.06.037 29986184

[221] W.‐C. HuangFu , M.‐W. Chao , C.‐C. Cheng , et al., “Anti‐leukemia Effects of the Novel Synthetic 1‐benzylindole Derivative 21‐900 in Vitro and in Vivo,” Scientific Reports 7 (2017): 42291. 10.1038/srep42291 PMC5299419 28181578

[222] S. Boichuk , A. Galembikova , F. Bikinieva , et al., “2‐APCAs, the Novel Microtubule Targeting Agents Active against Distinct Cancer Cell Lines,” Molecules (Basel, Switzerland) 26, no. 3 (2021): 616. 10.3390/molecules26030616 PMC7865999 33503939

[223] A. Al Absi , H. Wurzer , C. Guerin , et al., “Actin Cytoskeleton Remodeling Drives Breast Cancer Cell Escape from Natural Killer‐Mediated Cytotoxicity,” Cancer Research 78, no. 19 (2018): 5631–5643. 10.1158/0008-5472.CAN-18-0441 30104240

[224] Y. Zhang , M. Wang , L. Ye , et al., “HKDC1 promotes Tumor Immune Evasion in Hepatocellular Carcinoma by Coupling Cytoskeleton to STAT1 Activation and PD‐L1 Expression,” Nature Communications 15, no. 1 (2024): 1314. 10.1038/s41467-024-45712-2 PMC10864387 38351096

[225] L. M. Yin and M. Schnoor , “Modulation of Membrane–cytoskeleton Interactions: Ezrin as Key Player,” Trends in Cell Biology 32, no. 2 (2022): 94–97. 10.1016/j.tcb.2021.09.005 34625363

[226] M. Tameishi , H. Ishikawa , C. Tanaka , et al., “Ezrin Contributes to the Plasma Membrane Expression of PD–L1 in A2780 Cells,” Journal of Clinical Medicine 11, no. 9 (2022): 2457. 10.3390/jcm11092457 PMC9100183 35566582

[227] T. Shi , Y. Zhang , Y. Wang , et al., “DKK1 Promotes Tumor Immune Evasion and Impedes Anti–PD‐1 Treatment by Inducing Immunosuppressive Macrophages in Gastric Cancer,” Cancer Immunology Research 10, no. 12 (2022): 1506–1524. 10.1158/2326-6066.CIR-22-0218 36206576

[228] Y. Wei , S. Long , M. Zhao , et al., “Regulation of Cellular Signaling with an Aptamer Inhibitor to Impede Cancer Metastasis,” Journal of the American Chemical Society 146, no. 1 (2024): 319–329. 10.1021/jacs.3c09091 38129955

[229] Y. Wang , Y. Liu , L. Wang , et al., “Combretastatin A4‐Based Albumin Nanoparticles Remodeling the Tumor Immune Microenvironment to Enhance T Cell Immunotherapy in Colon Cancer,” ACS Nano 19, no. 26 (2025): 23746–23759. 10.1021/acsnano.5c03638 40553481

[230] V. Saxena , P. Patil , P. Khodke , and B. V. Kumbhar , “Exploring Purine Analogues as Inhibitors against Katanin, a Microtubule Severing Enzyme Using Molecular Modeling Approach,” Scientific Reports 14, no. 1 (2024): 32095. 10.1038/s41598-024-83723-7 PMC11686324 39738711

[231] Y. Luo , B. Lu , Y. Hu , et al., “Triptonoterpene Promotes the Mitochondrial Translocation of Cofilin‐1 to Induce Apoptosis in Gastric Cancer Cells by Regulating Actin Dynamics,” Biochemical Pharmacology 241 (2025): 117173. 10.1016/j.bcp.2025.117173 40675226

[232] X. Yu , K. Ballard , C. Collier , X. Wei , S. Ning , and C. Wang , “Targeting and Disrupting Cytoskeleton Using Core‐shell Metal‐organic Framework Nanoparticles to Inhibit Cancer Cell Migration,” Journal of Colloid and Interface Science 704, no. Pt 1 (2026): 139298. 10.1016/j.jcis.2025.139298 41129942

[233] G. P. Pathak , R. Shah , B. E. Kennedy , et al., “RTN4 Knockdown Dysregulates the AKT Pathway, Destabilizes the Cytoskeleton, and Enhances Paclitaxel‐Induced Cytotoxicity in Cancers,” Molecular Therapy 26, no. 8 (2018): 2019–2033. 10.1016/j.ymthe.2018.05.026 PMC6094397 30078441

[234] M. Beljkas , M. Petkovic , A. Vuletic , et al., “Development of Novel ROCK Inhibitors via 3D‐QSAR and Molecular Docking Studies: a Framework for Multi‐Target Drug Design,” Pharmaceutics 16, no. 10 (2024): 1250. 10.3390/pharmaceutics16101250 PMC11514586 39458584

[235] Z. Cao , X. Wei , S. Xing , et al., “Discovery of Novel Pyrazolo[1,5‐a]Pyrimidine Derivatives as Selective ROCK2 Inhibitors with Anti‐breast Cancer Migration and Invasion Activities,” Bioorganic Chemistry 151 (2024): 107675. 10.1016/j.bioorg.2024.107675 39126868

[236] N. Hemavathy , V. Umashankar , and J. Jeyakanthan , “Unveiling Novel Type 1 Inhibitors for Targeting LIM Kinase 2 (LIMK2) for Cancer Therapeutics: an Integrative Pharmacoinformatics Approach,” Computational Biology and Chemistry 115 (2025): 108289. 10.1016/j.compbiolchem.2024.108289 39631222

[237] R. Hedna , A. DiMaio , M. Robin , et al., “2‐Aminothiazole‐Flavonoid Hybrid Derivatives Binding to Tau Protein and Responsible for Antitumor Activity in Glioblastoma,” International Journal of Molecular Sciences 24, no. 20 (2023): 15050. 10.3390/ijms242015050 PMC10606064 37894731

[238] A. Yamauchi , A. Kobayashi , H. Oikiri , and Y. Yokoyama , “Functional Role of the Tau Protein in Epithelial Ovarian Cancer Cells,” Reproductive Medicine and Biology 16 (2017): 143–151. 10.1002/rmb2.12019 PMC5661812 29259462

[239] M. S. Ong , S. Deng , C. E. Halim , et al., “Cytoskeletal Proteins in Cancer and Intracellular Stress: a Therapeutic Perspective,” Cancers 12, no. 1 (2020): 238. 10.3390/cancers12010238 PMC7017214 31963677

[240] S. Ogata , K.‐I. Morishige , K. Sawada , et al., “Fasudil Inhibits Lysophosphatidic Acid‐induced Invasiveness of human Ovarian Cancer Cells,” International Journal of Gynecological Cancer 19, no. 9 (2009): 1473–1480. 10.1111/IGC.0b013e3181c03909 19955921

[241] C. Prunier , V. Josserand , J. Vollaire , et al., “LIM Kinase Inhibitor Pyr1 Reduces the Growth and Metastatic Load of Breast Cancers,” Cancer Research 76, no. 12 (2016): 3541–3552. 10.1158/0008-5472.CAN-15-1864 27216191

[242] J. Ginés , E. Sabater , C. Martorell , et al., “Efficacy of Taxanes as Adjuvant Treatment of Breast Cancer: a Review and Meta‐analysis of Randomised Clinical Trials,” Clinical & Translational Oncology: Official Publication of the Federation of Spanish Oncology Societies and of the National Cancer Institute of Mexico 13, no. 7 (2011): 485–498. 10.1007/s12094-011-0686-x 21775276

[243] M. C. Wani , H. L. Taylor , M. E. Wall , P. Coggon , and A. T. McPhail , “Plant Antitumor Agents. VI. Isolation and Structure of Taxol, a Novel Antileukemic and Antitumor Agent from Taxus Brevifolia,” Journal of the American Chemical Society 93, no. 9 (1971): 2325–2327. 10.1021/ja00738a045 5553076

[244] S. Ramalingam and C. P. Belani , “Taxanes for Advanced Non‐small Cell Lung Cancer,” Expert Opinion on Pharmacotherapy 3, no. 12 (2002): 1693–1709. 10.1517/14656566.3.12.1693 12472367

[245] Z. Ma , W. Zhang , B. Dong , et al., “Docetaxel Remodels Prostate Cancer Immune Microenvironment and Enhances Checkpoint Inhibitor‐based Immunotherapy,” Theranostics 12, no. 11 (2022): 4965–4979. 10.7150/thno.73152 PMC9274752 35836810

[246] C. Dumontet and M. A. Jordan , “Microtubule‐binding Agents: a Dynamic Field of Cancer Therapeutics,” Nature Reviews Drug Discovery 9, no. 10 (2010): 790–803. 10.1038/nrd3253 PMC3194401 20885410

[247] V. Čermák , V. Dostál , M. Jelínek , et al., “Microtubule‐targeting Agents and Their Impact on Cancer Treatment,” European Journal of Cell Biology 99, no. 4 (2020): 151075. 10.1016/j.ejcb.2020.151075 32414588

[248] M. L. Madsen , H. Due , N. Ejskjær , P. Jensen , J. Madsen , and K. Dybkær , “Aspects of Vincristine‐induced Neuropathy in Hematologic Malignancies: a Systematic Review,” Cancer Chemotherapy and Pharmacology 84, no. 3 (2019): 471–485. 10.1007/s00280-019-03884-5 PMC6682573 31214762

[249] C. Gourmelon , H. Bourien , P. Augereau , A. Patsouris , J.‐S. Frenel , and M. Campone , “Vinflunine for the Treatment of Breast Cancer,” Expert Opinion on Pharmacotherapy 17, no. 13 (2016): 1817–1823. 10.1080/14656566.2016.1217991 27484180

[250] S. N. Hansen , D. Westergaard , M. B. H. Thomsen , et al., “Acquisition of Docetaxel Resistance in Breast Cancer Cells Reveals Upregulation of ABCB1 Expression as a Key Mediator of Resistance Accompanied by Discrete Upregulation of Other Specific Genes and Pathways,” Tumor Biology 36, no. 6 (2015): 4327–4338. 10.1007/s13277-015-3072-4 25596703

[251] C. J. Paller and E. S. Antonarakis , “Cabazitaxel: a Novel Second‐line Treatment for Metastatic Castration‐resistant Prostate Cancer,” Drug Design, Development and Therapy 5, no. 10 (2011): 117–124. 10.2147/DDDT.S13029 PMC3063116 21448449

[252] X. Pivot , P. Koralewski , J. L. Hidalgo , et al., “A Multicenter Phase II Study of XRP6258 Administered as a 1‐h i.v. infusion every 3 Weeks in Taxane‐resistant Metastatic Breast Cancer Patients,” Annals of Oncology 19, no. 9 (2008): 1547–1552. 10.1093/annonc/mdn171 18436520

[253] W. Krause , “Resistance to Anti‐tubulin Agents: from Vinca Alkaloids to Epothilones,” Cancer Drug Resistance (Alhambra, Calif) 2, no. 1 (2019): 82–106. 10.20517/cdr.2019.06 PMC9019178 35582143

[254] I. Ramos , K. Stamatakis , C. L. Oeste , and D. Pérez‐Sala , “Vimentin as a Multifaceted Player and Potential Therapeutic Target in Viral Infections,” International Journal of Molecular Sciences 21, no. 13 (2020): 4675. 10.3390/ijms21134675 PMC7370124 32630064

